# Water Structuring Induces Nonuniversal Hydration Repulsion between Polar Surfaces: Quantitative Comparison between Molecular Simulations, Theory, and Experiments

**DOI:** 10.1021/acs.langmuir.3c03656

**Published:** 2024-04-05

**Authors:** Alexander Schlaich, Jan O. Daldrop, Bartosz Kowalik, Matej Kanduč, Emanuel Schneck, Roland R. Netz

**Affiliations:** † Stuttgart Center for Simulation Science (SC SimTech), University of Stuttgart, 70569 Stuttgart, Germany; ‡ Institute for Computational Physics, University of Stuttgart, 70569 Stuttgart, Germany; § Fachbereich Physik, Freie Universität Berlin, Arnimallee 14, 14195 Berlin, Germany; ∥ Department of Theoretical Physics, Jožef Stefan Institute, SI-1000 Ljubljana, Slovenia; ⊥ Institut für Physik Kondensierter Materie, Technische Universität Darmstadt, Hochschulstrasse 8, Darmstadt 64289, Germany

## Abstract

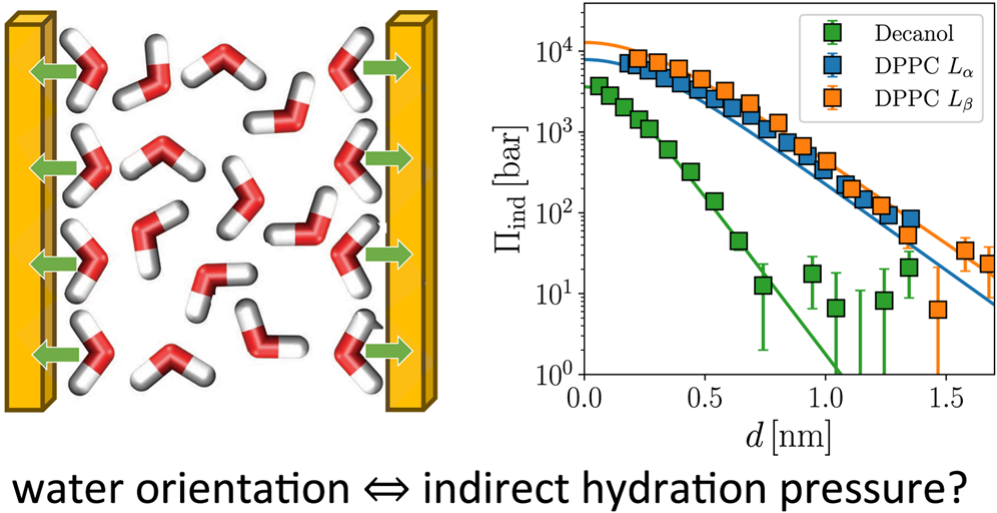

Polar surfaces in water typically repel each other at close separations, even if they are charge-neutral. This so-called hydration repulsion balances the van der Waals attraction and gives rise to a stable nanometric water layer between the polar surfaces. The resulting hydration water layer is crucial for the properties of concentrated suspensions of lipid membranes and hydrophilic particles in biology and technology, but its origin is unclear. It has been suggested that surface-induced molecular water structuring is responsible for the hydration repulsion, but a quantitative proof of this water-structuring hypothesis is missing. To gain an understanding of the mechanism causing hydration repulsion, we perform molecular simulations of different planar polar surfaces in water. Our simulated hydration forces between phospholipid bilayers agree perfectly with experiments, validating the simulation model and methods. For the comparison with theory, it is important to split the simulated total surface interaction force into a direct contribution from surface–surface molecular interactions and an indirect water-mediated contribution. We find the indirect hydration force and the structural water-ordering profiles from the simulations to be in perfect agreement with the predictions from theoretical models that account for the surface-induced water ordering, which strongly supports the water-structuring hypothesis for the hydration force. However, the comparison between the simulations for polar surfaces with different headgroup architectures reveals significantly different decay lengths of the indirect water-mediated hydration-force, which for laterally homogeneous water structuring would imply different bulk-water properties. We conclude that laterally inhomogeneous water ordering, induced by laterally inhomogeneous surface structures, shapes the hydration repulsion between polar surfaces in a decisive manner. Thus, the indirect water-mediated part of the hydration repulsion is caused by surface-induced water structuring but is surface-specific and thus nonuniversal.

## Introduction

The hydration force is a repulsive interaction between polar yet charge-neutral surfaces in water that dominates other interactions at surface separations below about 2 nm. It was discussed by Langmuir already in 1938,^1^ and analogous forces are also present in nonaqueous solvents.^2−4^ The hydration force prevents the tight adhesion of uncharged polar objects in an aqueous solution and, thus, plays an important role in maintaining a high level of hydration and fluidity in biological and colloidal systems. It is therefore rightfully considered a fundamental force in aqueous systems^5,6^ and determines the behavior of many industrially and biologically relevant systems, such as the stability of colloidal dispersions^7^ and soap films,^8,9^ the swelling of clays,^10^ and the interactions between biological membranes^11^ and between macromolecules.^12^ Pressure–distance measurements on net-neutral multilamellar stacks of phospholipid bilayers, which constitute a perfect model system to study the hydration forces, showed that the hydration repulsion decays approximately exponentially as a function of the membrane separation with typical decay lengths between 0.1 and 0.3 nm.^13−16^ In fact, the decay length exhibits significant variation even for identical systems depending on the precise definition of the membrane separation.^17^

Later studies^18−21^ suggested that the decay length of the hydration force is similar to the size of a water molecule for a wide class of interacting macromolecular systems in an aqueous solution, including DNA double helices, stiff polysaccharides, and proteins,^12^ and that the hydration force is not caused primarily by surface interactions but rather is due to some type of water structuring and thus might be universal. However, the precise molecular mechanism causing the hydration force still eludes quantitative theoretical explanation.^22^ In fact, a recent careful comparison of the experimentally measured hydration repulsion between phospholipid bilayers in the gel and liquid phases revealed significantly different decay lengths,^17^ regardless of whether the repeat distance in the lamellar stacks or the separation between the membrane surfaces was used in the exponential fit. This finding clearly contradicts the idea that the hydration force decays identically for different surface types.

The splitting of the total force acting between polar surfaces in water into the contribution from direct molecular interaction between the surface groups, called the direct force, and the rest, called the indirect force and which is due to the response of the water to the presence of the surfaces, is revealing the following (of course this splitting is only possible for molecular simulations): it turns out that for phospholipid bilayers, the direct force is attractive while the indirect force is repulsive and that they have very similar magnitudes.^17,23−25^ So the experimentally measured hydration force results from the almost complete cancellation of the competing direct and indirect force contributions. The fact that the resulting total hydration force is repulsive is by no means self-evident and rather hinges on a subtle balance between the direct and indirect force contributions, which in turn is primarily determined by the magnitude of the surface polarity.^26^ Since the direct force is mostly due to the electrostatic interactions between the polar groups on the surfaces,^25^ its magnitude and decay length depend sensitively on the distribution and dipolar strength of the polar groups on the surface. Accordingly, it is highly specific to the surface structure, and the direct contribution to the hydration force can therefore not be universal but rather depends on structural surface details. Thus, there is no reason why the hydration force, which receives substantial contributions from the direct force, should be universal. In contrast, the analysis of the indirect hydration force from simulations, which excludes direct membrane–membrane interactions, yielded very similar decay lengths for gel and liquid phospholipid bilayers,^17^ a fact that is completely masked in experimental measurements of the hydration force. So, if there is universality in the hydration force, it can only be found in its indirect contribution. The matter is further complicated by the fact that the definition of membrane separation, which can be taken, e.g., as the bilayer-stack repeat distance or the water-slab thickness, influences the decay length.^17^

Three fundamentally different mechanisms for the repulsive hydration force have been proposed in literature, namely, (i) repulsion due to the release of the surface-bound water molecules as the surfaces approach,^27,28^ (ii) steric repulsion between the membrane lipids and reduction of their configurational entropy,^29,30^ and (iii) repulsion due to the destructive interference of the structured interfacial water layers.^31^ Obviously, mechanisms (i) and (iii) are, according to our splitting of the total hydration force, indirect force contributions, while mechanism (ii) would be assigned to the direct hydration force if one neglects the influence of hydration water on the steric lipid repulsion. Since the direct hydration force in simulations of lipid bilayers is found to be attractive,^25^ mechanism (ii) cannot be a general model for the overall repulsive nature of the hydration force since also systems without significant changes in the configurational entropy upon variation of surface separation exhibit hydration repulsion. Mechanism (i) was found to act only at extremely short separation, when the last hydration layers are removed.^25^ Thus, in this paper, we concentrate on mechanism (iii).

A first attempt to rationalize the indirect contribution to the hydration repulsion based on water structuring was developed in the late 1970s by Marčelja and Radić,^31^ who formulated a Gaussian mean-field model for an unspecified water structural order parameter with fixed surface values in the spirit of a Landau–Ginzburg model. This model has been further refined and is frequently interpreted in terms of water-dipole orientational ordering and the nonlocal dielectric water response.^32−35^ It has been noted by Ninham that the relevant water ordering could also be related to the tetrahedrality or changes in the hydrogen-bond network.^36^ In fact, the description of the electrostatic and structural interactions in polarizable liquids based on continuum theory is currently regaining significant interest.^37−43^ Although such theoretical models provide conceptual insight and scaling laws for the proposed structural force, what is lacking in the literature is a quantitative comparison of the predicted water-structure profiles and indirect hydration-force magnitudes with real molecular systems. Experimentally, this is difficult because of the presence of the competing direct hydration interactions and because water-structure profiles are experimentally not available for varying surface separations. This is where molecular simulations come in, which have continuously improved since the early days of lipid molecular dynamics (MD) simulations.^44,45^ In fact, we have recently developed methods to perform simulations of confined water at a prescribed water chemical potential,^25,46^ which is the relevant ensemble for many experiments and applications and which allowed us to quantitatively compare the hydration pressures for lipid bilayer systems between MD simulations and experiments.^17,25,47^

**Figure 1:**
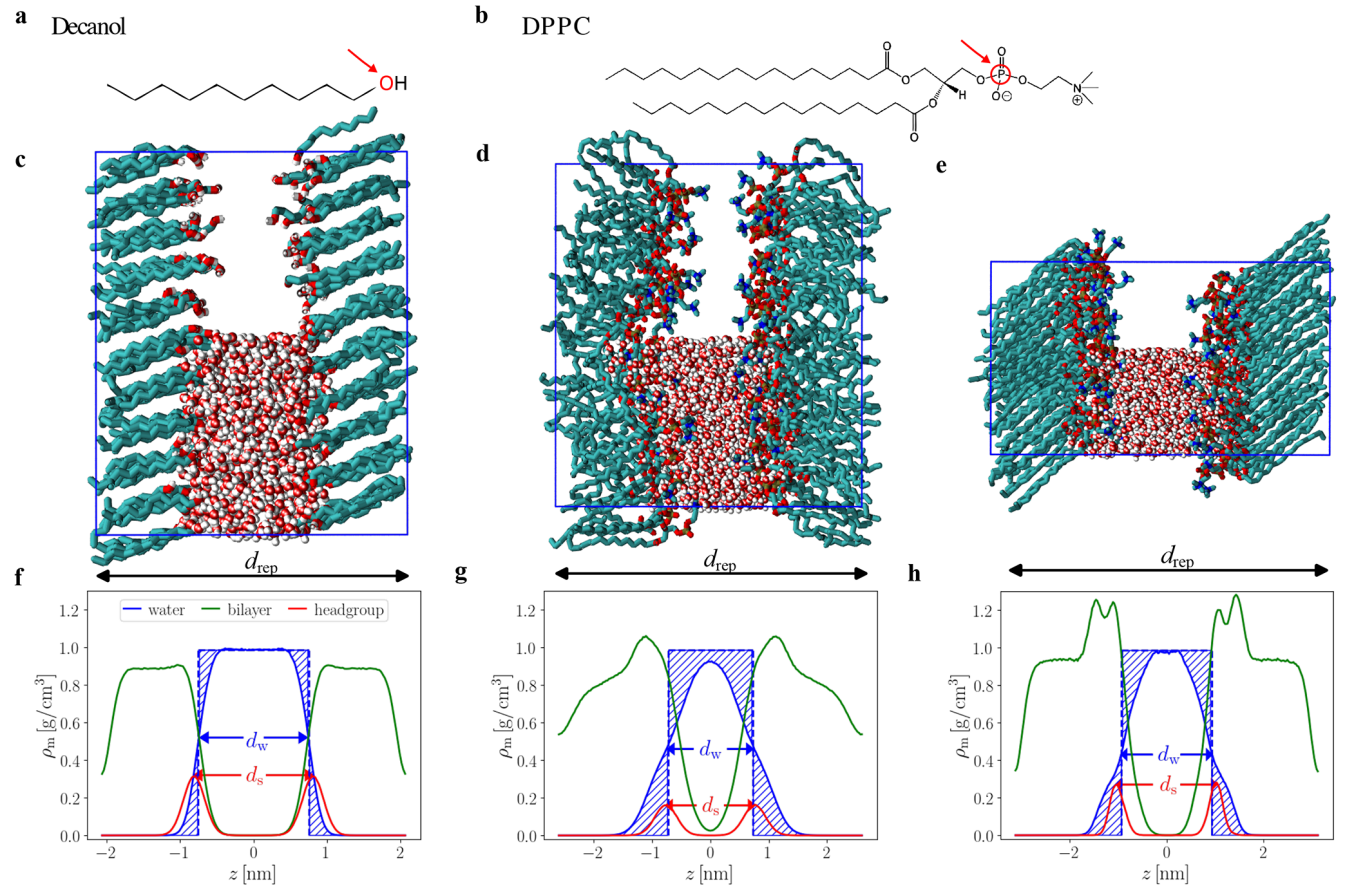
Simulation setup: our atomistic simulations resolve the chemical structures of the surfaces consisting of (a) decanol or (b) DPPC molecules and include explicit water. Simulation snapshots for (c) decanol, (d) liquid-phase DPPC, and (e) gel-phase DPPC bilayers. For clarity, water is only shown in the lower half of the periodically repeated simulation box indicated by the blue rectangle. Red arrows in (a,b) indicate the headgroup atoms selected for defining the structural surface separation *d*_s_. (f–h) show the corresponding mass density profiles for water (blue lines), lipids or decanols (green lines), and the selected headgroup atoms (red lines). The repeat distance *d*_rep_, the water-slab thickness *d*_w_, and the structural distance *d*_s_ are indicated. Corresponding values are *d*_rep_ = (4.1, 5.2, 6.2) nm for the systems in (f–h), respectively. Vertical blue dashed lines and shaded areas indicate the construction of the Gibbs-dividing surfaces from which the water-slab thickness *d*_w_ is determined. From *d*_s_ the surface separation *d* used in our quantitative analysis of the hydration force is derived, as explained in the text.

In this paper, we analyze simulations of interacting hydroxyl-terminated bilayers composed of grafted decanol molecules (see Figure 1c for a simulation snapshot) as well as dipalmitoylphosphatidylcholine (DPPC) bilayers in the disordered *L*_α_ liquid and the ordered *L*_β_ gel state (see Figure 1d,e, respectively). We note that decanol when dispersed in water does not form a lamellar phase,^48,49^ we rather employ our surfaces composed of grafted decanols as a generic model for strongly polar surfaces such as self-assembled monolayers made from long-chain alcohols. DPPC was chosen for our simulations since it is ubiquitous in biological systems,^50^ its phase behavior has been amply studied,^51^ and reliable and consistent experimental results for the hydration repulsion are available.^18,52−55^ Our simulations employ atomistic models for the surfaces and for the water that include electrostatic, steric, as well as van der Waals interactions. The total interaction forces between the phospholipid bilayers extracted from our simulations agree perfectly with experimental results, which validates the simulation methods and models. The simulated water-structure profiles and indirect water-mediated hydration forces are compared quantitatively with predictions from a simple Landau–Ginzburg model and are in agreement with earlier work.^56^ This without a doubt confirms that the surface-induced water structuring causes the hydration repulsion between the polar surfaces. We compare different order parameters as descriptors of the water structure, such as the electric polarization or multipole densities, and demonstrate that the surface-induced water structure is complex and highly surface-specific. All simulation details are given in Section I of the Supporting Information; we here only note that our conclusions are rather independent of the choice of force-field or water model, which demonstrates the robustness of the mechanism causing the hydration force (see our comparison of simulations with different force-fields in Section II of the Supporting Information).

The comparison of three different polar surface types is insightful as it allows us to demonstrate that the decay length of the indirect hydration force is not universal but depends on the surface type. Since the decay length according to the Landau–Ginzburg model only depends on bulk water properties, we conclude that the Landau–Ginzburg model, in its simplest one-dimensional formulation, although it perfectly describes the water ordering and hydration force profiles, misses an essential feature. We argue that the assumption of a laterally averaged scalar order-parameter profile is too restrictive and that the water structure that actually causes the hydration force is laterally inhomogeneous and shaped by the lateral surface structure, as has been suggested before.^57−59^ We conclude that water-structuring causes the indirect part of the hydration repulsion between the polar surfaces, but the specific surface structure plays a non-negligible role and influences not only the magnitude but also the decay length of this contribution to the total hydration repulsion. Thus, even the indirect contribution to the hydration repulsion is nonuniversal and surface-specific.

## Materials and Methods

### Hydration Pressure and Water Structuring from the Landau–Ginzburg Model

In their pioneering work,^31^ Marčelja and Radić predicted an exponential decay of the hydration repulsion caused by the structural properties of water confined between two parallel planar surfaces. According to their model, the water perturbation due to the polar surfaces is described in terms of an order-parameter profile; the effects of the surfaces enter via boundary conditions or surface fields. If one assumes translational invariance parallel to the surfaces, an assumption we critically discuss in this paper, then a one-dimensional mean-field model is obtained.

According to the Landau approach,^60^ the water-structure-dependent free-energy density is written as an expansion in terms of the scalar order parameter , which only depends on the position normal to the surfaces. To the lowest order one obtains for the free energy per area *A* rescaled by the inverse thermal energy β = 1/*k*_B_*T*^31^
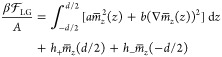
1where surface effects are included via surface fields *h*_±_ that couple linearly to the order parameter  at the interfaces located at *z* = *d*/2 and *z* = −*d*/2.^33^ The volume contribution (i.e., what is inside the integral in eq 1) can be derived by a long-wavelength expansion of the free energy of a dipolar fluid (see Sections III and IV in the Supporting Information), where *a* and *b* are positive parameters which determine the variance and spatial correlations of , respectively. Care has to be taken when defining the surface boundary conditions, since switching from a fixed surface order-parameter boundary condition (as in the original work by Marčelja and Radić)^31^ to a fixed surface-field boundary condition changes the sign of the interaction pressure.^56,61^ We will later demonstrate that the constant surface-field boundary condition in eq 1 agrees very well with the simulation results and is therefore the correct one. In Section V of the Supporting Information, we show that the linear surface coupling (in the two terms outside of the integral in eq 1) is the special case of a more general expression that includes a quadratic coupling to the surface order parameter, from which the two different surface boundary conditions can by obtained by a suitable limiting procedure.^62^ In the present work, we not only primarily associate the order parameter  with the laterally averaged electric polarization density normal to the surfaces but also discuss higher-order multipole densities and their gradients as candidates for the relevant structural order parameter.

The mean order-parameter profile follows from eq 1 by variational minimization, yielding
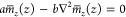
2
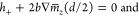
3
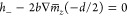
4

At the surface, the water molecules have a preferred orientation due to their interactions with the polar surface groups and the interfacial hydrogen-bonding structure.^63^ If  corresponds to the normal polarization and the surfaces are identical, we have *h*_+_ = −*h*_–_ ≡ *h* by symmetry, and the order-parameter profile is antisymmetric with respect to the symmetry plane in the middle of the water slab at *z* = 0, i.e.,  (in Section VI of the Supporting Information, we present the results for the quadrupole density as an example for an order parameter that exhibits a symmetric profile between identical surfaces).

Defining , the antisymmetric solution of the linear second-order differential eq 2 is given by
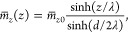
5where we have defined the correlation length λ = (*b*/*a*)^1/2^. Combining eqs 3–5, we obtain the polarization at the surface as
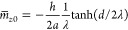
6The free energy eq 1 then follows as^33^
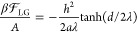
7from which the indirect contribution to the normal pressure is obtained via a derivative with respect to the separation as
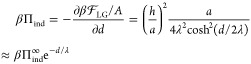
8

This pressure is repulsive and decays exponentially for large distances *d* ≫ λ with an amplitude βΠ_ind_^∞^ = *h*^2^/(*a*λ^2^) = *h*^2^/*b*. Note again that eq 8 corresponds to the indirect, water-mediated part of the total hydration pressure since the free energy in eq 1 does not include the direct surface–surface interactions. Importantly, for symmetric order-parameter profiles, the interaction pressure is in fact attractive, as shown in Sections V and VI of the Supporting Information, which means that only order parameters that exhibit antisymmetric profiles are suitable candidates to explain the hydration repulsion under the fixed surface-field boundary condition.

## Results and Discussion

### Comparison of Simulated Hydration Pressure with Experimental Data

When one compares simulation results with experiments or theory, as mentioned above and as previously noted,^17,56^ the proper and consistent definition of the membrane separation is crucial but not unique. In this work, we define the surface separation *d* in the simulations based on the mean structural distance *d*_s_ between the oxygen atoms in the opposing layers for decanol and between the phosphorus atoms in the opposing DPPC layers, respectively, as indicated in Figure 1. From *d*_s_ we subtract the mean value at zero water content *d*_s_^0^ according to *d* = *d*_s_ – *d*_s_^0^, so that the resulting *d* is zero in the absence of hydration water. We obtain values *d*_s_^0^ = 0.27 nm for the decanol system and *d*_s_^0^ = 0.46 nm for DPPC in both liquid and gel states.

This structural definition of the surface separation *d* improves the agreement between the simulated polarization profiles and their surface values with the theoretical predictions compared with the alternative typical definition based on the water slab thickness *d*_w_, as discussed in Section VII of the Supporting Information. In analogy to experiments,^14^*d*_w_ is defined as *d*_w_ = *N*_w_*v*_w_/*A*, where *v*_w_ = 0.0304 nm^3^ is the bulk molecular water volume and *N*_w_ is the number of water molecules confined between the surfaces of lateral area *A*. The superior performance of our definition of *d* when comparing simulations with theory is not unexpected: while *d*_w_ is the thermodynamic definition of the water slab thickness based on the Gibbs dividing surface positions, the surface separation *d* in the Landau–Ginzburg model reflects the position where the surfaces couple to the water polarization (see Section VII in the Supporting Information for a discussion). In contrast, when simulations are compared with experiments, *d*_w_ is the preferred distance definition because it can be consistently derived in simulations and experiments.

**Figure 2:**
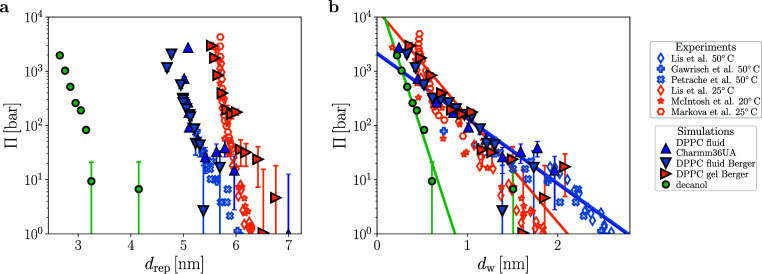
Total hydration pressures between DPPC membranes and decanol bilayers. Simulated (filled symbols) and experimental data (open symbols) are shown as a function of (a) the periodic repeat distance *d*_rep_ and (b) the water slab thickness *d*_w_. For liquid-phase and gel-phase DPPC bilayers, data for the hydration pressure Π from simulations and experiments are compared; for decanol, only simulation data are shown. The solid lines in (b) show exponential fits to the simulation data with decay lengths  for liquid DPPC (blue),  for gel DPPC (orange), and  for decanol (green). Experimental data are taken from refs (18,52−55) and converted to different distance scales according to ref (17). The united atom description of the lipid tails in the simulations results in an underestimation of *d*_rep_, therefore the simulation data for DPPC in (a) are shifted by 2 Å for the liquid and by 5 Å for the gel phase. Data reproduced with permission from refs (52 and 54) copyright 1982 and 1992 Elsevier and from ref (18), copyright 1998 American Physical Society.

In Figure 2, we show the equivalent hydration pressure Π for DPPC in the osmotic stress ensemble, where the hydrostatic pressure is fixed at Π_osm_ = 1 bar. This is an ensemble used in many experiments on multilamellar stacks of bilayers. In (a), we compare simulation with experimental results as a function of the lamellar repeat distance *d*_rep_ and in (b) as a function of the water slab thickness *d*_w_. In the experiments, the DPPC lamellar spacing is varied by subjecting the water to an osmotic stress. In the simulations, we measure the water chemical potential μ_osm_(*N*_w_) as described in refs (25 and 46) and Section VIII of the Supporting Information and in analogy to the experimental procedure convert this chemical potential to an equivalent hydrostatic pressure according to
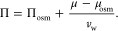
9Here, Π is the hydrostatic pressure predicted to act between the bilayers at normal-state chemical potential μ, based on the corresponding ambient conditions Π_osm_ = 1 bar and μ_osm_, which is the chemical potential exerted in the osmotic stress experiment. Since water is nearly incompressible, in the conversion, one can use the constant *v*_w_ = 0.0304 nm^3^ for the water molecular volume in bulk at a pressure of 1 bar, as obtained from separate simulations. Note that eq 9 follows to first order in Π – Π_osm_ from the Gibbs–Duhem equation, see Section VIII in the Supporting Information for details.

Comparison of the simulations (triangles, data taken from ref (17)) with experimental data in the gel and liquid states of the DPPC membranes taken from refs (18,52−55) shows excellent agreement for both distance definitions. Exponential fits to the simulation data in Figure 2b yield decay lengths  for the gel and  for the liquid DPPC bilayers, shown as orange and blue solid lines, respectively (in the fits, pressure data that are negative due to numerical noise are excluded).^17^ The tilde indicates that the decay lengths are defined by using the water slab thickness *d*_w_. Corresponding fits to the experimental pressures yield decay lengths  for gel and  for liquid DPPC membranes, in very good agreement with the simulation data (the fits to the experimental data are shown in ref (17)).

In Figure 2, we include simulation results for the decanol bilayers taken from ref (64) as green circles. Due to the positional restraints present in this model system (see Section I in the Supporting Information), simulations in the *N*_w_Π*T* ensemble are not straightforwardly possible. Instead, we perform simulations at a constant box volume *V* = *AL*_*z*_ and measure the chemical potential μ at fixed *L*_*z*_ for different water numbers *N*_w_. By interpolation, we determine the pressure at the water chemical potential in bulk under normal conditions Π(μ) shown in Figure 2. We find an exponential decay length of , indicated by the solid green line in Figure 2b, which is much shorter than the decay lengths in liquid and gel DPPC layers. The different decay lengths are not caused by the different ensembles used for the decanol and DPPC bilayer simulations, as we have demonstrated previously and will corroborate in the next section.^65^ We thus find that the decay length of the total hydration pressure depends on the surface type and is not universal.^17^ We mention in passing that our simulation results do not depend on details of the simulation parameters and the chosen force-fields, as demonstrated in Section II of the Supporting Information.

### Decomposition of Simulated Hydration Pressure into Direct and Indirect Contributions

**Figure 3:**
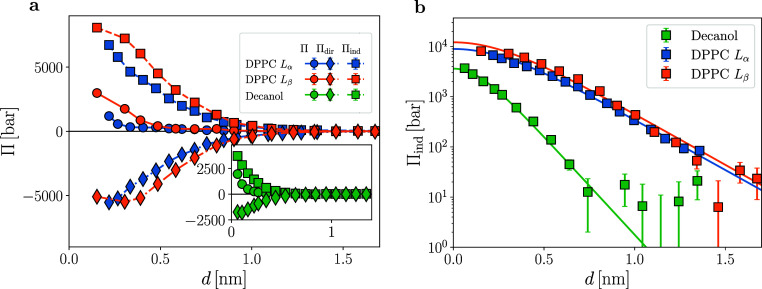
Decomposition of hydration pressure: (a) decomposition of the simulated total hydration pressure Π (circles) into the direct membrane–membrane contribution Π_dir_ (diamonds) and the indirect water-mediated contribution Π_ind_ (squares). Lines are guides to the eye, the inset shows the decanol data separately. (b) Indirect pressures (symbols) with fits to the Landau–Ginzburg pressure given by eq 8 (lines). The corresponding decay lengths λ_Πind_ are given in Table 1.

In order to be able to decompose the hydration pressure Π into the direct and indirect parts according to Π = Π_dir_ + Π_ind_, we perform simulations of the DPPC bilayer systems at varying hydrostatic pressures that are adjusted to yield water chemical potentials corresponding to the bulk value under normal conditions (see Sections VIII and IX in the Supporting Information for details). For decanol, we use our simulations where the water number *N*_w_ is adjusted such that the chemical potential equals the corresponding normal-condition value. Both simulation ensembles thus correspond to the experimental scenario where the water is in equilibrium with a pure water bulk phase, and thus, the water chemical potential between the surfaces is the same as in bulk. The pressure decomposition in Figure 3a shows that for all systems, the direct pressure Π_dir_ is strongly attractive, whereas the indirect water-mediated pressure Π_ind_ is repulsive and overcompensates for the direct attraction, giving rise to a repulsive total pressure Π. The strong attraction in the direct pressure directly rules out a possible explanation for the hydration repulsion in terms of direct interactions.^25^ The near-cancellation of the direct and indirect contributions at large separations has been discussed recently^17^ and is expected from electrostatic considerations: The direct attraction is mainly electrostatic in nature and due to Coulomb attraction between opposite charges in the polar head groups.^25^ On a simplistic level, the water polarization reduces this attraction to about 1/ε ≈ 0.01 of its value in vacuum, where ε denotes the bulk water dielectric permittivity. This simple electrostatic consideration, with a homogeneous dielectric constant that is assumed independent of the surface separation, already shows that the direct and indirect contributions must compensate to a large degree; yet, this simple argument would result in an attractive total pressure and therefore cannot explain the hydration repulsion, so a purely electrostatic interpretation with a bulk-like dielectric permittivity does not do justice to the hydration repulsion.

The indirect pressures are shown in Figure 3b on a logarithmic scale; they can be perfectly fitted to the Landau–Ginzburg prediction in eq 8 shown as solid lines. The resulting fit values for the decay length λ_Πind_ are given in Table 1. Notably, there is only a negligible difference between the liquid and gel DPPC membranes, whereas the decay length for the decanol bilayers is shorter by a factor of about two. So we conclude that even for the indirect hydration pressure contribution, which we might expect to be universal and determined by water properties alone, different surfaces are characterized by vastly different decay lengths, which we will further discuss below.

**Table 1:** Simulation Fit Values^a^

	decanol	DPPC *L*_α_	DPPC *L*_β_	bulk
λ_Πind_ [nm]	0.11	0.22	0.21	
λ_*m̅*_z__ [nm]	0.14	0.28	0.25	
λ_*m̅*_z__(1) [nm]	0.13	0.27	0.24	
λ_*m̅*_z__(2) [nm]	0.14	0.25	0.21	
*h*/*a* [e/nm]	–0.011	–0.273	–0.329	
*h*^(1)^/*a*^(1)^ [e/nm]	0.027	–0.228	–0.285	
*h*^(2)^/*a*^(2)^ [e/nm]	–0.040	0.037	–0.037	
*a* [nm/e^2^]	35,684	492	496	355 (300 K)/323 (330 K)
*a*^(1)^ [nm/e^2^]	6047	706	661	
*a*^(2)^ [nm/e^2^]	2755	26,791	39,222	

a The decay lengths λ_Πind_ are obtained from fits of eq 8 to the simulated indirect pressure in Figure 3b, and the decay lengths , , and  are obtained from fits of eq 5 to the simulated polarization and its dipolar and quadrupole contributions in Figure 4. The rescaled surface fields *h*/*a*, *h*^(1)^/*a*^(1)^, and *h*^(2)^/*a*^(2)^ are obtained from fits of eq 6 to the simulation data in Figure 5. The order-parameter stiffness values *a*, *a*^(1)^, and *a*^(2)^ in the first three columns are obtained from fits of eq 8 to the pressure simulation data in Figure 3b. The value in the last column is derived from the bulk-water dielectric permittivity as shown in Section V of the Supporting Information.

### Comparison of Simulated Order-Parameter Profiles with Landau–Ginzburg Model Predictions

There are infinitely many different order parameters that can be used to characterize the water structure between polar surfaces. This can be appreciated by splitting the perpendicular polarization profile  into its multipole contributions according to
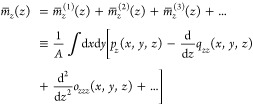
10Here, the dipolar density *p*_*z*_(*x*, *y*, *z*), the quadrupolar density *q*_*zz*_(*x*, *y*, *z*), and the octupolar density *o*_*zzz*_(*x*, *y*, *z*) are defined in terms of the *z*-components of the multipole expansion  of the charge distribution of molecule *i* with partial charges *q*_*j*_^*i*^ at positions **r**_*j*_^*i*^ up to the third moment, respectively. The laterally averaged density of the *z*-component of the dipolar density *p*_*z*_(*x*, *y*, *z*) is denoted by ,  is the laterally averaged gradient of the *zz*-component of the quadrupolar density *q*_*zz*_(*x*, *y*, *z*), and  is the laterally averaged curvature of the *zzz*-component of the octupole density *o*_*zzz*_(*x*, *y*, *z*) (higher-order contributions are negligible). For the reference position **r**_*i*_ in each molecule, we choose the water oxygen. As water has no net charge, the dipole moment (*l* = 1 in the multipole expansion) is independent of the reference position, whereas the higher-order multipoles depend on this choice. Assuming these contributions to  to be independent order parameters, they could all contribute to the hydration force, as we will examine further below.

In Figure 4, we show profiles of the polarization and its dipolar and quadrupolar contributions for the three considered systems at different surface separations *d*. The octupole moment makes a rather small contribution to the polarization, as is discussed in Section X of the Supporting Information. All profiles can be fitted nicely to the Landau–Ginzburg prediction given in eq 5 and shown as solid lines. The resulting fit values for , , and  are given in Table 1 and agree well the with the decay lengths λ_Πind_ obtained from the indirect pressure fits, which is a testimony to the consistency of the Landau–Ginzburg model. Figure 4a–c shows the results for the decanol bilayer system. Strikingly, one observes a near-cancellation of the dipolar profile in (b) and the quadrupolar profile in (c), leading to relatively weak polarization in (a) whose sign is dominated by the quadrupolar contribution; to appreciate this, one has to compare the absolute value of  to  and  in (b) and (c). This is very different for the liquid and gel phospholipid bilayers shown in Figure 4d–i, where the magnitudes of the quadrupolar profiles are rather small and thus the polarization  and dipolar density profiles  are very similar.

**Figure 4:**
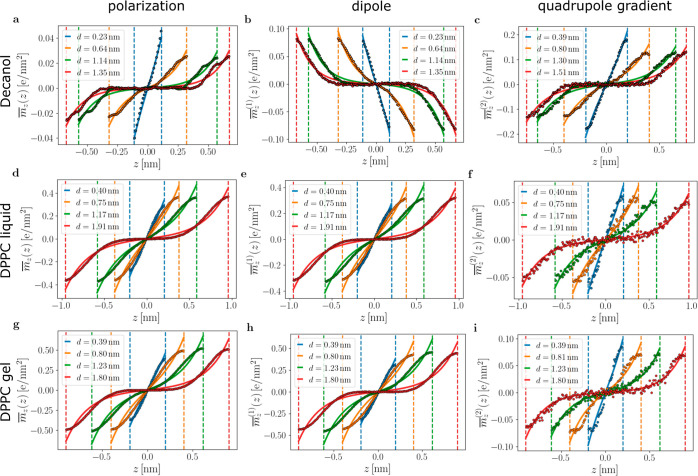
Water polarization profiles: simulation data for (a–c) decanol bilayers, (d–f) DPPC bilayers in the disordered *L*_α_ phase, and (g–i) DPPC bilayers in the ordered *L*_β_ phase. The first column shows the polarization , the second column the dipolar contribution , and the third column the quadrupolar contribution  to the polarization. The dashed vertical lines indicate the surface positions at ± *d*/2, the corresponding values of *d* are given in the legends. Solid lines are fits according to eq 5 leading to decay lengths λ given in Table 1. The surface order-parameter values  extracted from the fits are shown in Figure 5.

The different signs of the dipolar density profiles in Figure 4 for DPPC and decanol bilayers point to opposite water orientations. The negative dipolar density at the lower DPPC layer for *z* < 0 means that water hydrogens point toward the lipid phase. This is at first sight surprising, since the zwitterionic lipid headgroup has an opposite dipolar orientation with the positively charged choline group being closer to the water phase than the negatively charged phosphate group. The reason for this is that the interfacial water molecules are in fact inside the headgroup and located between the choline and phosphate charges, as demonstrated in nonlinear optics experiments and simulations.^66^ Using the SPC/E dipole moment *P*_0_ = 4.893 × 10^–2^ e·nm, full orientation in bulk water would give a dipolar density of . Compared to this maximally possible value, the dipolar densities at the DPPC surface in Figure 4e,h reveal a rather high degree of orientation, in particular considering that the water density decreases as one moves toward the lipid phase. We conclude that the zwitterionic DPPC headgroup strongly orients the interfacial water, which is due to the complex headgroup chemistry and presumably involves water hydrogen-bonding effects. The different sign of the dipolar density at the decanol surface in Figure 4b is related to the fact that the decanol headgroup dipole is dominated by the negative partial charge on the oxygen and positive partial charges on the carbon atoms and thus oriented oppositely compared to that in DPPC. Since the dipole moment of a decanol molecule is significantly smaller compared to that of a DPPC molecule, the water dipolar density  at the decanol surface is much smaller compared to that at the DPPC surface. It can be speculated that the DPPC headgroup is evolutionarily engineered to strongly orient water and thus give rise to a strong hydration repulsion. Quadrupolar and octupolar density profiles can also be nicely fitted to the Landau–Ginzburg model, but note that the quadrupolar density profile is symmetric and thus gives rise to an attractive hydration force for a constant surface field boundary condition; see Section III in the Supporting Information for details.

**Figure 5:**
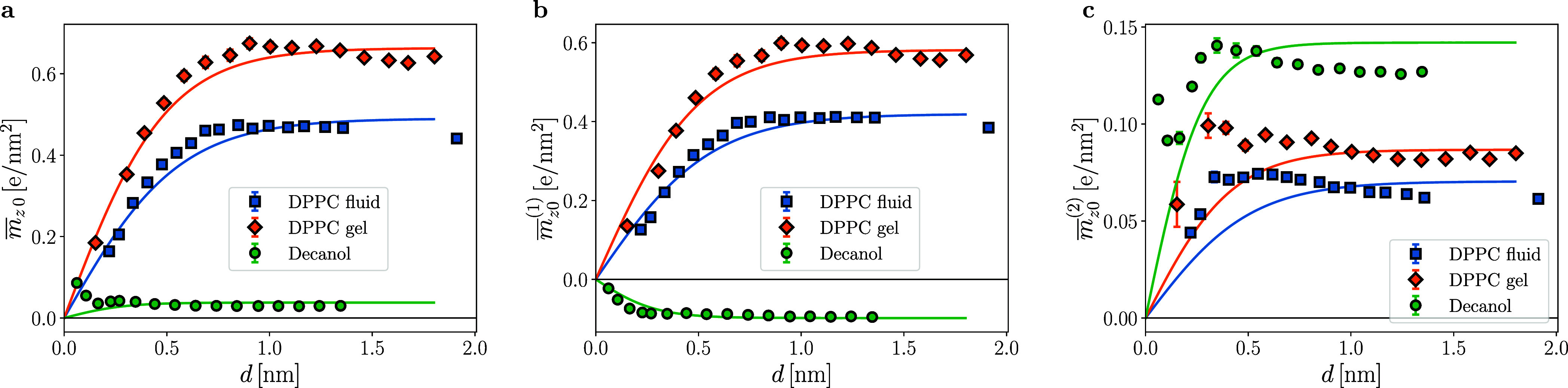
Order-parameter surface values: the data for  are obtained from the fits of the polarization profiles to eq 5 in Figure 4. Results are shown for (a) the polarization , (b) the dipolar contribution , and (c) the quadrupolar contribution . Solid lines denote eq 6 where the amplitudes *h*/*a* are fitted to the simulation data, while the corresponding correlation lengths λ are taken from the fits in Figure 4 and given in Table 1.

In Figure 5a, we show the polarization surface values  (extracted from the fits in Figure 4) as a function of separation *d* for the three studied systems. The DPPC results are well described by the Landau–Ginzburg model prediction eq 6 (solid lines), revealing that a constant surface field *h*, as assumed in eq 1, is the correct boundary condition.^56^ The decanol polarization data in Figure 5a are not well described by eq 6, which is not surprising as the polarization results from near-cancellation between the competing dipolar and quadrupolar contributions, as seen in Figure 4. The dipolar surface densities in Figure 5b are all well described by eq 6 discussed before; the water dipoles at DPPC and decanol surfaces point in opposite directions. For the surface densities of the quadrupolar gradient in Figure 5c, we see pronounced deviations from the model predictions; one also observes that for decanol, the quadrupole contribution is larger than that for DPPC. Via the fits of eq 6 to the data shown in Figure 5 we determine the ratio *h*/*a*, which is given for the polarization and its multipole contributions in Table 1.

We have now determined all parameters appearing in the Landau–Ginzburg model, namely, the rescaled surface-field strength *h*/*a*, obtained from the fits of eq 6 to the simulated surface order-parameter values in Figure 5, the decay length λ = (*b*/*a*)^1/2^, and the order-parameter stiffness *a*, obtained from fits of eq 8 to the simulated indirect pressures in Figure 3b, all given in Table 1. The comparison of the Landau–Ginzburg model predictions with the simulation data looks generally favorable, and the fit values for the decay length λ are consistent between the pressure and order-parameter data. The rescaled surface-field strength *h*/*a* in Table 1 is different for the different systems and also for different order parameters, which is expected. The decay length λ = (*b*/*a*)^1/2^ and the order-parameter stiffness parameter *a*, given in Table 1, are different for the decanol and DPPC systems. This is unexpected since the parameters *a* and *b* are bulk water parameters that should not depend on the surface type.

We have added in Table 1 the stiffness value *a* that follows from the bulk-water dielectric permittivity, determined by bulk-water polarization fluctuations, as derived in Section V of the Supporting Information. The bulk-water value for *a* is rather close to the results for DPPC, but it deviates strongly from the results for decanol. The deviation between the bulk prediction and the values in the decanol and DPPC systems for *a* is not entirely unexpected, since it is known that polarization fluctuations perpendicular to surfaces in confined systems are much reduced compared to those in bulk,^64^ but the significant deviation among the different confined systems studied here is puzzling.

The polarization in the decanol system results from the near cancellation of the dipolar and quadrupolar contributions, as shown in Figure 4, which indicates that the polarization might not be the best possible order parameter for decanol. Therefore, in Table 1, we also show the stiffness of the dipolar polarization contribution *a*^(1)^ and the stiffness of the quadrupolar polarization contribution *a*^(2)^ obtained from fits of eq 8 to the simulation data for the indirect hydration pressure in Figure 3b using the results for the rescaled surface-field strengths *h*^(1)^/*a*^(1)^ and *h*^(2)^/*a*^(2)^ given in Table 1. These fits use the hypothetical assumption that the hydration pressure is exclusively produced by water structuring that corresponds to either the dipolar or the quadrupolar polarization contribution. We observe that for DPPC, the polarization stiffness *a* and the dipolar stiffness *a*^(1)^ are roughly the same, which reverberates that for DPPC, the polarization is mostly due to its dipolar contribution, as shown in Figure 4. However, the dipolar stiffness *a*^(1)^ differs vastly between decanol and DPPC, which is perplexing even when considering that for decanol, the dipolar order parameter will presumably be coupled to the quadrupolar order parameter in some way.

We conclude that the Landau–Ginzburg model describes the polarization profiles of water in Figure 4 and the indirect hydration pressures in Figure 3b convincingly well, yet a comparison of the order-parameter stiffness parameter *a* among different systems and the bulk value reveals a substantial and unexpected disagreement, in particular for decanol. Something seems to be missing in the one-dimensional Landau–Ginzburg model.

We have so far considered laterally averaged order-parameter profiles that are a function of *z* only. Assuming that the ordering is described by the polarization, due to lateral isotropy of the planar system the order parameter is scalar and corresponds to the polarization in the *z*-direction. It is fairly straightforward to relax this restriction: in Section XI of the Supporting Information, we derive the Landau–Ginzburg model for a vectorial polarization order parameter that is laterally modulated from the nonlocal density functional theory for a dipolar fluid. The results show that the laterally modulated polarization components, which are missed when laterally averaging over the order-parameter profiles, are non-negligible and presumably play an important role for hydration interactions and can explain the observed discrepancies of the Landau–Ginzburg parameter values between the different systems we extract from our simulations: The effective *a* and *b* parameters of the Landau–Ginzburg model for a vectorial polarization order parameter in Section XI of the Supporting Information depend on the lateral modulation wave vector, which plausibly is different for different surfaces. Indeed, in line with this hypothesis, in Section XII of the Supporting Information, we show that the lateral components of water dipoles at opposing decanol surfaces are correlated in molecular simulations. Clearly, more work in this direction is needed.

## Conclusions

To unravel the mechanism that causes the hydration repulsion between polar surfaces, we perform molecular simulations of three different planar polar surface types in water. Our simulated hydration forces between fluid and gel phospholipid bilayers agree perfectly with experiments, which validates our simulation model and methods.

To check whether surface-induced water structuring produces hydration repulsion, we reconsider the phenomenological Landau–Ginzburg model for a one-dimensional scalar order parameter between two symmetric surfaces with opposite surface fields, which produces an antisymmetric order-parameter profile and leads to a repulsive interaction. The predictions of this model agree very nicely with the profiles of the laterally averaged perpendicular polarization, , its multipolar contributions, and the indirect part of the hydration force from molecular simulations of all three different hydrated polar systems, namely, decanol and liquid and gel DPPC bilayers. By fits of the model predictions to the simulation data, the three parameters of the Landau–Ginzburg model are uniquely extracted for each system.

It turns out that the parameters of the Landau–Ginzburg model that describe bulk water exhibit vastly different values for different systems. This signals an inconsistency in the one-dimensional Landau–Ginzburg model, in particular, for the decanol system, since the bulk-water properties should not depend on the surface type.

There are several possible reasons for this inconsistency: one possibility is that we have simply missed the correct water structuring order parameter (although it should be noted that we have considered quite a few possible order parameters, as we discuss below). Another possibility is the neglect of the finite width of the surface coupling to the water structural order parameter.^67,68^ As an alternative explanation, we suggest that the hydration pressure between polar surfaces might, for some surfaces, be associated with water structuring that involves a laterally modulated polarization that points in the lateral direction. Interestingly, these polarization contributions can be described by an identical Landau–Ginzburg model but with bulk parameters that depend on the lateral modulation wave vector, as we show in Section XI of the Supporting Information. As support for this hypothesis, we show in Section XII of the Supporting Information that in our molecular simulations, the dipolar components on opposing decanol surfaces that are parallel to the surfaces are correlated. Taken together, this suggests that for some surfaces, a significant contribution to the hydration pressure might stem from water structural correlations that are modulated laterally and involve polarization ordering parallel to the surfaces. We conclude that water structuring significantly contributes to the hydration repulsion between polar surfaces, although the actual order parameter that describes the water structuring depends on the specific surface structure. Consequently, the indirect contribution to the hydration repulsion between polar surfaces is nonuniversal and depends on the surface type.

As mentioned before, water structuring can be described by different order parameters. Only antisymmetric order-parameter profiles give rise to repulsive forces in the presence of linear surface coupling. This crucially limits the possible candidates for order parameters that could explain the hydration repulsion. Besides the multipolar contributions to the polarization, which are the dipole density and gradients of the higher-order multipole densities, further candidates are the antisymmetric higher multipole densities such as the octupole density (which is the third moment), the fifth moment, and seventh moment. However, we find that already the octupole orientation contributes negligibly to the repulsion (see Section VI in the Supporting Information for details). So, we conclude that the order parameters based on the electric polarization and its leading multipolar contributions that include lateral modulation effects might be worthwhile to pursue further in future work on hydration interactions.

## Supporting Information Available

All simulation details, comparison with the Charmm36UA force-field, derivation of the Landau free energy with generalized boundary conditions, nonlocal density-functional theory for polarizable fluids, mapping between Landau–Ginzburg model and nonlocal polarization theory, quadrupole and octupole order-parameter profiles, details on the distance definition, thermodynamic extrapolation method and the decomposition of the interaction pressure, results from the multipole expansion of the polarization density, laterally inhomogeneous order-parameter profiles, and analysis of lateral dipolar correlations between water molecules (PDF)

## References

[ref1] Langmuir I. The Role of Attractive and Repulsive Forces in the Formation of Tactoids, Thixotropic Gels, Protein Crystals and Coacervates. J. Chem. Phys. 1938, 6, 873–896. 10.1063/1.1750183.

[ref2] Persson P. K.; Bergenståhl B. Repulsive forces in lecithin glycol lamellar phases. Biophys. J. 1985, 47, 743–746. 10.1016/S0006-3495(85)83974-6. 4016195 PMC1435184

[ref3] Christenson H. K.; Horn R. G. Solvation forces measured in non-aqueous liquids. Chem. Scr. 1985, 25, 37–41.

[ref4] Kuznetsova N.; Rau D. C.; Parsegian V. A.; Leikin S. Solvent hydrogen-bond network in protein self-assembly: solvation of collagen triple helices in nonaqueous solvents. Biophys. J. 1997, 72, 353–362. 10.1016/S0006-3495(97)78674-0. 8994620 PMC1184324

[ref5] Mahanty J. F.; Ninham B. W. Dispersion Forces; Academic Press: London-New York-San Francisco, 1976; Vol. 81.

[ref6] Parsegian V. A. Van Der Waals Forces: A Handbook for Biologists, Chemists, Engineers, and Physicists; Cambridge University Press, 2005.

[ref7] Dubois M.; Schönhoff M.; Meister A.; Belloni L.; Zemb T.; Möhwald H. Equation of state of colloids coated by polyelectrolyte multilayers. Phys. Rev. E 2006, 74, 051402 10.1103/PhysRevE.74.051402. 17279904

[ref8] Clunie J. S.; Goodman J. F.; Symons P. C. Solvation Forces in Soap Films. Nature 1967, 216, 1203–1204. 10.1038/2161203a0.

[ref9] Mysels K. J. Solvation Forces in Soap Films. Nature 1968, 218, 265–266. 10.1038/218265a0.

[ref10] Pashley R. M.; Quirk J. P. The effect of cation valency on DLVO and hydration forces between macroscopic sheets of muscovite mica in relation to clay swelling. Colloids Surf. 1984, 9, 1–17. 10.1016/0166-6622(84)80138-9.

[ref11] Lipowsky R. The conformation of membranes. Nature 1991, 349, 475–481. 10.1038/349475a0. 1992351

[ref12] Stanley C.; Rau D. C. Evidence for water structuring forces between surfaces. Curr. Opin. Colloid Interface Sci. 2011, 16, 551–556. 10.1016/j.cocis.2011.04.010. 22125414 PMC3223916

[ref13] Israelachvili J. N.; Adams G. Measurement of forces between two mica surfaces in aqueous electrolyte solutions in the range 0–100 nm. J. Chem. Soc., Faraday Trans. 1 1978, 74, 975–1001. 10.1039/f19787400975.

[ref14] Parsegian V.; Fuller N.; Rand R. Measured work of deformation and repulsion of lecithin bilayers. Proc. Natl. Acad. Sci. U.S.A. 1979, 76, 2750–2754. 10.1073/pnas.76.6.2750. 288063 PMC383686

[ref15] Rand R.; Parsegian V. Hydration forces between phospholipid bilayers. Biochim. Biophys. Acta 1989, 988, 351–376. 10.1016/0304-4157(89)90010-5.

[ref16] Marsh D. Water adsorption isotherms and hydration forces for lysolipids and diacyl phospholipids. Biophys. J. 1989, 55, 1093–1100. 10.1016/S0006-3495(89)82906-6. 2765647 PMC1330575

[ref17] Kowalik B.; Schlaich A.; Kanduč M.; Schneck E.; Netz R. R. Hydration Repulsion Difference between Ordered and Disordered Membranes Due to Cancellation of Membrane–Membrane and Water-Mediated Interactions. J. Phys. Chem. Lett. 2017, 8, 2869–2874. 10.1021/acs.jpclett.7b00977. 28590133

[ref18] Petrache H. I.; Gouliaev N.; Tristram-Nagle S.; Zhang R.; Suter R. M.; Nagle J. F. Interbilayer interactions from high-resolution x-ray scattering. Phys. Rev. E 1998, 57, 7014–7024. 10.1103/PhysRevE.57.7014.

[ref19] Petrache H. I.; Zemb T.; Belloni L.; Parsegian V. A. Salt screening and specific ion adsorption determine neutral-lipid membrane interactions. Proc. Natl. Acad. Sci. U.S.A. 2006, 103, 7982–7987. 10.1073/pnas.0509967103. 16702553 PMC1461405

[ref20] Aroti A.; Leontidis E.; Dubois M.; Zemb T. Effects of Monovalent Anions of the Hofmeister Series on DPPC Lipid Bilayers Part I: Swelling and In-Plane Equations of State. Biophys. J. 2007, 93, 1580–1590. 10.1529/biophysj.106.094482. 17496051 PMC1948043

[ref21] Schneck E.; Demé B.; Gege C.; Tanaka M. Membrane Adhesion via Homophilic Saccharide-Saccharide Interactions Investigated by Neutron Scattering. Biophys. J. 2011, 100, 2151–2159. 10.1016/j.bpj.2011.03.011. 21539782 PMC3149267

[ref22] Parsegian V.; Zemb T. Hydration forces: Observations, explanations, expectations, questions. Curr. Opin. Colloid Interface Sci. 2011, 16, 618–624. 10.1016/j.cocis.2011.06.010.

[ref23] Pertsin A.; Platonov D.; Grunze M. Origin of Short-Range Repulsion between Hydrated Phospholipid Bilayers: A Computer Simulation Study. Langmuir 2007, 23, 1388–1393. 10.1021/la0622929. 17241063

[ref24] Eun C.; Berkowitz M. L. Origin of the Hydration Force: Water-Mediated Interaction between Two Hydrophilic Plates. J. Phys. Chem. B 2009, 113, 13222–13228. 10.1021/jp901747s. 19518117

[ref25] Schneck E.; Sedlmeier F.; Netz R. R. Hydration repulsion between biomembranes results from an interplay of dehydration and depolarization. Proc. Natl. Acad. Sci. U.S.A. 2012, 109, 14405–14409. 10.1073/pnas.1205811109. 22908241 PMC3437872

[ref26] Kanduč M.; Netz R. R. From Hydration Repulsion to Dry Adhesion between Asymmetric Hydrophilic and Hydrophobic Surfaces. Proc. Natl. Acad. Sci. U.S.A. 2015, 112, 12338–12343. 10.1073/pnas.1504919112. 26392526 PMC4603451

[ref27] Israelachvili J. N.; Wennerström H. Role of hydration and water structure in biological and colloidal interactions. Nature 1996, 379, 219–225. 10.1038/379219a0. 8538786

[ref28] Wennerström H.; Sparr E. Thermodynamics of membrane lipid hydration. Pure Appl. Chem. 2003, 75, 905–912. 10.1351/pac200375070905.

[ref29] Israelachvili J. N.; Wennerstroem H. Hydration or steric forces between amphiphilic surfaces?. Langmuir 1990, 6, 873–876. 10.1021/la00094a028.

[ref30] Israelachvili J. N.; Wennerstroem H. Entropic forces between amphiphilic surfaces in liquids. J. Phys. Chem. 1992, 96, 520–531. 10.1021/j100181a007.

[ref31] Marčelja S.; Radić N. Repulsion of interfaces due to boundary water. Chem. Phys. Lett. 1976, 42, 129–130. 10.1016/0009-2614(76)80567-2.

[ref32] Radić N.; Marčelja S. Solvent contribution to the debye screening length. Chem. Phys. Lett. 1978, 55, 377–379. 10.1016/0009-2614(78)87043-2.

[ref33] Cevc G.; Podgornik R.; Zeks B. The free energy,enthalpy and entropy of hydration of phospholipid bilayer membranes and their difference on the interfacial separation. Chem. Phys. Lett. 1982, 91, 193–196. 10.1016/0009-2614(82)83639-7.

[ref34] Ruckenstein E.; Schiby D. On the origin of repulsive hydration forces between two mica plates. Chem. Phys. Lett. 1983, 95, 439–443. 10.1016/0009-2614(83)80590-9.

[ref35] Attard P.; Batchelor M. T. A mechanism for the hydration force demonstrated in a model system. Chem. Phys. Lett. 1988, 149, 206–211. 10.1016/0009-2614(88)87223-3.

[ref36] Ninham B. W. Long-range vs. short-range forces. The present state of play. J. Phys. Chem. 1980, 84, 1423–1430. 10.1021/j100449a001.

[ref37] de Souza J.; Kornyshev A. A.; Bazant M. Z. Polar Liquids at Charged Interfaces: A Dipolar Shell Theory. J. Chem. Phys. 2022, 156, 244705 10.1063/5.0096439. 35778078

[ref38] Berthoumieux H.; Monet G.; Blossey R. Dipolar Poisson Models in a Dual View. J. Chem. Phys. 2021, 155, 024112 10.1063/5.0056430. 34266284

[ref39] Monet G.; Bresme F.; Kornyshev A.; Berthoumieux H. Nonlocal Dielectric Response of Water in Nanoconfinement. Phys. Rev. Lett. 2021, 126, 216001 10.1103/PhysRevLett.126.216001. 34114838

[ref40] Blossey R.; Podgornik R. Field Theory of Structured Liquid Dielectrics. Phys. Rev. Res. 2022, 4, 023033 10.1103/PhysRevResearch.4.023033.

[ref41] Blossey R.; Podgornik R. Continuum Theories of Structured Dielectrics. Europhys. Lett. 2022, 139, 27002 10.1209/0295-5075/ac7d0a.

[ref42] Blossey R.; Podgornik R. A Comprehensive Continuum Theory of Structured Liquids. J. Phys. A: Math. Theor. 2023, 56, 025002 10.1088/1751-8121/acb40c.

[ref43] Hedley J. G.; Berthoumieux H.; Kornyshev A. A. The Dramatic Effect of Water Structure on Hydration Forces and the Electrical Double Layer. J. Phys. Chem. C 2023, 127, 8429–8447. 10.1021/acs.jpcc.3c00262.

[ref44] Marrink S. J.; Berkowitz M.; Berendsen H. J. C. Molecular dynamics simulation of a membrane/water interface: the ordering of water and its relation to the hydration force. Langmuir 1993, 9, 3122–3131. 10.1021/la00035a062.

[ref45] Essmann U.; Perera L.; Berkowitz M. L. The origin of the hydration interaction of lipid bilayers from MD simulation of dipalmitoylphosphatidylcholine membranes in gel and liquid crystalline phases. Langmuir 1995, 11, 4519–4531. 10.1021/la00011a056.

[ref46] Schlaich A.; Kowalik B.; Kanduč M.; Emanuel S.; Netz R. R. In Computational Trends in Solvation and Transport in Liquids; Sutmann G., Grotendorst J., Gompper G., Marx D., Eds.; IAS Series; Forschungszentrum Jülich GmbH: Jülich, 2015; Vol. 28; pp 155–185.

[ref47] Kanduč M.; Schlaich A.; de Vries A. H.; Jouhet J.; Maréchal E.; Demé B.; Netz R. R.; Schneck E. Tight cohesion between glycolipid membranes results from balanced water–headgroup interactions. Nat. Commun. 2017, 8, 14899 10.1038/ncomms14899. 28367975 PMC5382269

[ref48] Jokela P.; Joensson B. Phase Equilibria of Catanionic Surfactant-Dodecanol-Water Systems. J. Phys. Chem. 1988, 92, 1923–1927. 10.1021/j100318a043.

[ref49] Stephenson R.; Stuart J. Mutual Binary Solubilities: Water-Alcohols and Water-Esters. J. Chem. Eng. Data 1986, 31, 56–70. 10.1021/je00043a019.

[ref50] Stachowicz-Kuśnierz A.; Seidler T.; Rogalska E.; Korchowiec J.; Korchowiec B. Lung Surfactant Monolayer – A Good Natural Barrier against Dibenzo-p-Dioxins. Chemosphere 2020, 240, 124850 10.1016/j.chemosphere.2019.124850. 31561163

[ref51] Sparr E.; Wennerström H. Responding Phospholipid Membranes—Interplay between Hydration and Permeability. Biophys. J. 2001, 81, 1014–1028. 10.1016/S0006-3495(01)75759-1. 11463643 PMC1301571

[ref52] Lis L.; McAlister M.; Fuller N.; Rand R.; Parsegian V. Interactions between neutral phospholipid bilayer membranes. Biophys. J. 1982, 37, 657–665. 10.1016/S0006-3495(21)00385-4. 7074191 PMC1328851

[ref53] McIntosh T. J.; Magid A. D.; Simon S. A. Steric repulsion between phosphatidylcholine bilayers. Biochemistry 1987, 26, 7325–7332. 10.1021/bi00397a020. 3427075

[ref54] Gawrisch K.; Ruston D.; Zimmerberg J.; Parsegian V. A.; Rand R. P.; Fuller N. Membrane dipole potentials, hydration forces, and the ordering of water at membrane surfaces. Biophys. J. 1992, 61, 1213–1223. 10.1016/S0006-3495(92)81931-8. 1600081 PMC1260386

[ref55] Markova N.; Sparr E.; Wadsö L.; Wennerström H. A Calorimetric Study of Phospholipid Hydration. Simultaneous Monitoring of Enthalpy and Free Energy. J. Phys. Chem. B 2000, 104, 8053–8060. 10.1021/jp001020q.

[ref56] Kanduč M.; Schlaich A.; Schneck E.; Netz R. R. Hydration repulsion between membranes and polar surfaces: Simulation approaches versus continuum theories. Adv. Colloid Interface Sci. 2014, 208, 142–152. 10.1016/j.cis.2014.02.001. 24612664

[ref57] Kornyshev A. A.; Leikin S. Fluctuation theory of hydration forces: The dramatic effects of inhomogeneous boundary conditions. Phys. Rev. A 1989, 40, 6431–6437. 10.1103/PhysRevA.40.6431. 9902040

[ref58] Leikin S.; Kornyshev A. A. Theory of Hydration Forces. Nonlocal Electrostatic Interaction of Neutral Surfaces. J. Chem. Phys. 1990, 92, 6890–6898. 10.1063/1.458276.

[ref59] Leikin S.; Kornyshev A. A. Mean-field theory of dehydration transitions. Phys. Rev. A 1991, 44, 1156–1168. 10.1103/PhysRevA.44.1156. 9906065

[ref60] Landau L. Zur Theorie der phasenumwandlungen II. Phys. Z. Sowjetunion 1937, 11, 26–35.

[ref61] Schlaich A.; Kowalik B.; Kanduč M.; Schneck E.; Netz R. R. Physical mechanisms of the interaction between lipid membranes in the aqueous environment. Phys. A 2015, 418, 105–125. 10.1016/j.physa.2014.06.088.

[ref62] Podgornik R.; Žekš B. Hydration force and hydration regulation. Stud. Biophys. 1986, 111, 135–142.

[ref63] Bhide S. Y.; Berkowitz M. L. Structure and dynamics of water at the interface with phospholipid bilayers. J. Chem. Phys. 2005, 123, 224702–224702–16. 10.1063/1.2132277. 16375490

[ref64] Schlaich A.; Knapp E. W.; Netz R. R. Water Dielectric Effects in Planar Confinement. Phys. Rev. Lett. 2016, 117, 048001 10.1103/PhysRevLett.117.048001. 27494499

[ref65] Kanduč M.; Schneck E.; Netz R. R. Hydration Interaction between Phospholipid Membranes: Insight into Different Measurement Ensembles from Atomistic Molecular Dynamics Simulations. Langmuir 2013, 29, 9126–9137. 10.1021/la401147b. 23848998

[ref66] Dreier L. B.; Wolde-Kidan A.; Bonthuis D. J.; Netz R. R.; Backus E. H.; Bonn M. Unraveling the Origin of the Apparent Charge of Zwitterionic Lipid Layers. J. Phys. Chem. Lett. 2019, 10, 6355–6359. 10.1021/acs.jpclett.9b02587. 31568720

[ref67] Kirchner S.; Cevc G. Hydration of polar interfaces. A generalised mean-field model. J. Chem. Soc., Faraday Trans. 1994, 90, 1941–1951. 10.1039/ft9949001941.

[ref68] Cevc G.; Hauser M.; Kornyshev A. A. Effects of the Interfacial Structure on the Hydration Forces between Laterally Uniform Surfaces. Langmuir 1995, 11, 3103–3110. 10.1021/la00008a041.

