# Supplementary material for: Water Structuring Induces Nonuniversal Hydration Repulsion between Polar Surfaces: Quantitative Comparison between Molecular Simulations, Theory, and Experiments
Source: Langmuir. 2024 Apr 5;40(15):7896–906. doi: 10.1021/acs.langmuir.3c03656 (PMC11025125; doi:10.1021/acs.langmuir.3c03656)
Supplement: Supplementary file 1 — la3c03656_si_001.pdf [file la3c03656_si_001.pdf]

Supporting Information for:
Water Structuring Induces Nonuniversal Hydration Repulsion between Polar Surfaces: Quantitative Comparison between Molecular Simulations, Theory and Experiments

Alexander Schlaich,[1, 2] Jan O. Daldrop,[3] Bartosz Kowalik,[3]
Matej Kanduč,[4] Emanuel Schneck,[5] and Roland R. Netz[3, *]

[1] *Stuttgart Center for Simulation Science (SC SimTech), University of Stuttgart, 70569 Stuttgart, Germany*
[2] *Institute for Computational Physics, University of Stuttgart, 70569 Stuttgart, Germany*
[3] *Fachbereich Physik, Freie Universität Berlin, Arnimallee 14, 14195 Berlin, Germany*
[4] *Department of Theoretical Physics, Jožef Stefan Institute, SI-1000 Ljubljana, Slovenia*
[5] *Institut für Physik Kondensierter Materie, Technische Universität Darmstadt, Hochschulstrasse 8, Darmstadt, 64289, Germany*

CONTENTS

I. Simulation details 1
A. Decanol bilayer 2
B. Simulation of DPPC bilayers in the gel and fluid phase using the Berger forcefield 2

II. Comparison with DPPC simulations using the Charmm36UA force field 3

III. Non-local density-functional theory for polarizable fluids 4

IV. Mapping between Landau–Ginzburg model and non-local polarization theory 7

V. Landau free energy with generalized boundary conditions 7

VI. Quadrupole and octupole order-parameter profiles 9
A. Octupole order parameter 9
B. Quadrupole order parameter 11

VII. Distance definition 13

VIII. Thermodynamic extrapolation method 14

IX. Decomposition of the interaction pressure 14

X. Multipole expansion of the polarization density and factorized density-weighted dipole density 15

XI. Laterally inhomogeneous order-parameter profiles 16

XII. Lateral dipolar correlations between water molecules 17

References 18

I. SIMULATION DETAILS

All simulations are performed using the GROMACS package version 5.0 [1] for decanol and version 4.6 [2] for DPPC and are analyzed using our open-source software MAICoS (`https://www.maicos-analysis.org/`) based on the MDAnalysis package [3]. For all systems 3d-periodic boundary conditions and a time step of 2 fs are employed.

---

* rnetz@physik.fu-berlin.de

Lennard–Jones interactions are truncated at $r_c = 0.9\,\mathrm{nm}$, for the electrostatic interactions the Particle Mesh Ewald method [4] is employed with a real-space cut-off $r_c = 0.9\,\mathrm{nm}$.

As explained in detail below, simulations of decanol bilayers are performed at a water number determined such that the water chemical potential is equal to its value in bulk under standard conditions.. Simulations of DPPC bilayers are carried out in two different ensembles. In the first simulation setup, which mimics the so-called osmotic-stress ensemble that is used in many experiments, simulations are carried out at fixed water number and at a fixed normal pressure $\Pi_{\mathrm{osm}} = 1\,\mathrm{bar}$, here the hydration pressure Π is calculated from the measured chemical potential via the Gibbs–Duhem equation as explained below. In a second set of DPPC-bilayer simulations, the applied pressure Π is varied at fixed water number such that the water chemical potential is equal to its value in bulk under standard conditions, these simulations are used for decomposing the pressure into its direct and indirect contributions $\Pi = \Pi_{\mathrm{dir}} + \Pi_{\mathrm{ind}}$.

A. Decanol bilayer

For the decanol systems we simulate a bilayer consisting of two monolayers, each containing of $N_l = 100$ polar decanol $CH_3(CH_2)_9OH$ molecules. The periodic system is filled with a varying number of $N_w = 170$ up to 1,160 SPC/E water molecules [5]. Force-field parameters are based on GROMOS53A6 [6], where decanol hydroxyl groups are represented in atomistic detail, CH_2 and CH_3 groups as united atoms. The Lennard–Jones repulsion between head group oxygens is increased to reduce intra-surface hydrogen bonding [7]. To avoid slow reorientation events, we restrain all decanols on the second CH_2 group counting from the OH head group with force constants $k_x = k_y = 500\,\mathrm{kJ/(mol\,nm^2)}$ and $k_z = 10\,\mathrm{kJ/(mol\,nm^2)}$ and the terminal CH_3 group with $k_x = k_y = 5\,\mathrm{kJ/(mol\,nm^2)}$ on a centered rectangular lattice with a lateral area per head group of $A/N_l = 0.234\,\mathrm{nm}^2$ and decanol tilt angle of 30°, where the distance between opposing tails is chosen according to their equilibrium separation. Simulations are performed in the $N_w A L_z T$ ensemble at constant volume $V = AL_z$ and the number of water molecules N_w is adjusted via thermodynamic extrapolation to yield a constant chemical potential [8]. The extrapolation thus employs at least three sets of simulations at fixed L_z with different water numbers N_w. We use 18 simulations along the free energy perturbation reaction coordinate to obtain the chemical potential $\mu(N_w)$ using the MBAR method [9]. Each individual trajectory has a length of 100 ns, thus the total sampling time per data point in the pressure–distance curve for the decanol system is about 6 μs.

B. Simulation of DPPC bilayers in the gel and fluid phase using the Berger forcefield

For all DPPC simulation results shown in the main text we use the Berger lipid force field [10–12] and the SPC/E water model [5]. The assisted freezing method [13] is used for the construction of fully hydrated membranes in the L_β (gel) phase at a temperature of $T = 270\,\mathrm{K}$, controlled by the canonical velocity rescaling thermostat [14]. This fully hydrated membrane consists of 2×36 DPPC lipids hydrated by 40 water molecules per lipid. The structure is equilibrated at $T = 300\,\mathrm{K}$ and afterwards gradually dehydrated by removing one water molecule per lipid at a time and equilibration for 5 ns down to a hydration level of 3 waters per lipid molecule. All equilibration runs are performed in the $N\Pi T$ ensemble. To improve sampling, we use four different starting configurations, which are independently dehydrated five times with different random seeds, giving 20 different systems per hydration level. For production runs in the L_α fluid phase the temperature in the gel state is increased to 330 K, above the melting temperature of DPPC membranes in experiment and in simulations [15–18].

An anisotropic pressure coupling is employed using the Berendsen barostat [11] with a time constant of $\tau_P = 2\,\mathrm{ps}$. Each system is simulated for 5 ns, so the total simulation time is 100 ns per hydration level. In the osmotic ensemble, the pressure is set to $\Pi_{\mathrm{osm}} = 1\,\mathrm{bar}$ and the chemical potential μ_{osm} is measured. From the 20 different systems per hydration level the error of the chemical potential and thus the osmotic pressure is estimated, for which we perform 18 simulations along the free energy perturbation reaction coordinate. Prior to the production runs, fluid and gel membranes at all hydration levels are equilibrated for at least 5 ns and production runs are performed over another 5 ns, thus the total simulation time for the pressure–distance curves of DPPC exceeds 60 μs. To decompose the total interaction pressure into its direct and indirect parts as defined in the main manuscript, simulations in the hydrostatic ensemble at extrapolated pressures Π are performed [19]. We explicitly verified that the resulting chemical potential in this case equals the bulk water chemical potential.

II. COMPARISON WITH DPPC SIMULATIONS USING THE CHARMM36UA FORCE FIELD

The Berger parameters [11] are widely used for molecular dynamics simulations of lipid bilayers, yet excellent alternatives are available that perform better when compared to experimental observables such as the glycerol backbone structure [20]. In order to show that our results and conclusions are independent of the lipid forcefield and water model employed, we exemplarily perform simulations of DPPC bilayers using the united atom lipid chain version of CHARMM36 (C36-UA) [21] with the CHARMM version of the TIP3P water model [22]. We used the NAMD input files provided by Claire Loison for the NMRLipid projects (`https://zenodo.org/record/17004?ln=en`) and converted the topology using version 0.1.7 of the PyTopol script (`https://github.com/resal81/PyTopol`). Simulations for the lipid system using C36-UA were performed using a force-switch function for the Lennard–Jones interaction at 1.0 nm, whereas the short-range cut-off for the Coulomb interaction was set to 1.2 nm. The long-range electrostatics was computed using the Particle Mesh Ewald (PME) method [4]. The temperature in the C36-UA simulations was set to 323 K. All other simulation parameters agree with the aforementioned setup for fluid DPPC using the Berger forcefield. Simulations were performed using 5.1 of the GROMACS simulation package [1] and single point energies were checked against NAMD version 2.10 [23].

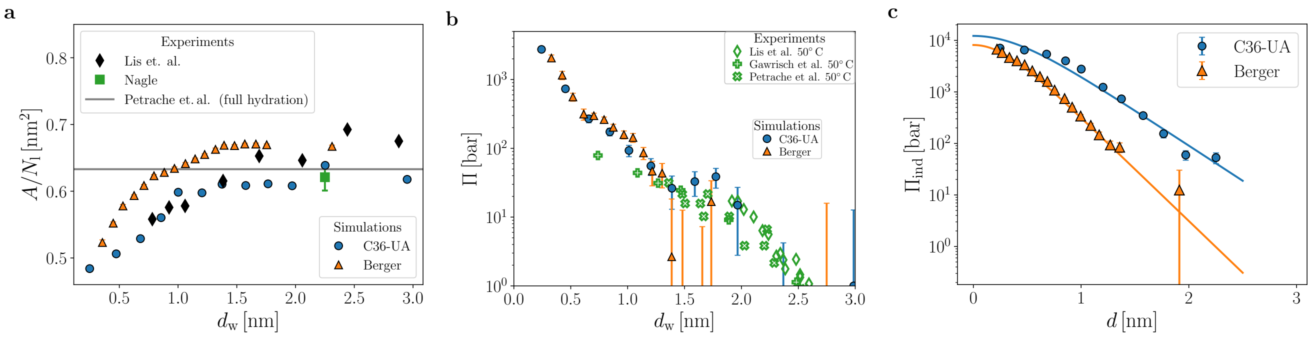

Figure S1. **Simulation results of DPPC in the liquid phase using the C36-UA force field.** (a) Area per lipid, A/N_l as function of the water slab thickness d_w. The simulation data for the two force-fields studied are compared to experimental data by Lis et al. [24], Nagle [25] and Petrache et al. [26]. (b) Comparison of the obtained pressure–distance curves in the osmotic stress ensemble for the two force-fields with experimental data from Refs. [24, 27, 28]. (c) Indirect simulation pressures (symbols) and fits to Eq. (8) of the main text (lines), with the decay length λ fixed by fits to the orientation profiles (shown in Fig. S2). As explained in the main text and below in Section IX, these simulations are performed at varying hydrostatic pressures that are adjusted to yield water chemical potentials corresponding to the bulk value at standard conditions.

Figure S1 (a) and (b) compare the simulation data using the C36-UA forcefield to the results using the Berger parameters, as employed in our analysis in the main text. The area per lipid, A/N_l in Fig. S1 (a) for C36-UA agrees slightly better with experimental values. However, both force fields reproduce the experimental reduction of lateral area upon dehydration and deviations are on the order of $\approx 5\%$. Strikingly, the pressure–distance curves for the two force fields in Fig. S1 (b) agree quantitatively with each other and the experimental values. This is at first surprising, as the indirect pressures in Fig. S1 (c) are significantly different both in amplitude and in decay length, but it confirms, independently of the employed forcefield, that direct (which are due to electrostatic and Van der Waals interactions between the bilayers) and indirect interactions (which are due to water-mediated forces) are well-balanced in phospholipid simulations, as has been found previously [29].

The decay length for the Landau–Ginzburg pressure, Eq. (8) shown in Fig. S1 (c) by the solid lines, is obtained from fits of the Landau–Ginzburg polarization profile Eq. (5) to the polarization profiles shown in Fig. S2 (a). The obtained value $\lambda_{\overline{m}_z}$ of 0.40 nm for C36-UA is significantly larger than 0.27 nm obtained for Berger in the main text. This difference is in fact expected considering the different water models: The bulk water dielectric constants differ significantly, $\varepsilon = 67$ for SPC/E at 330 K and $\varepsilon = 97$ for the CHARMM TIP3P model at 323 K, which we determined independently using the same simulation parameters as in the lipid system. As discussed in the main text, the dielectric constant in confinement differs significantly from bulk. Additionally, the detailed partial charge distribution in the head groups contributes significantly to the effective dielectric constant [30]. Thus, it is surprising that although the indirect interactions are significantly different in the two force fields (Fig. S1 (c)), the total pressures agree excellently (Fig. S1 (b)), which is due to the balance between water-mediated and direct, head group-head group interactions.

The perpendicular polarization profiles $\overline{m}_z(z)$ for C36-UA are shown in Fig. S2 (a) together with the fits of Eq. (5) in the main text to the data. Overall, the agreement between the simulated polarization profiles and the Landau–

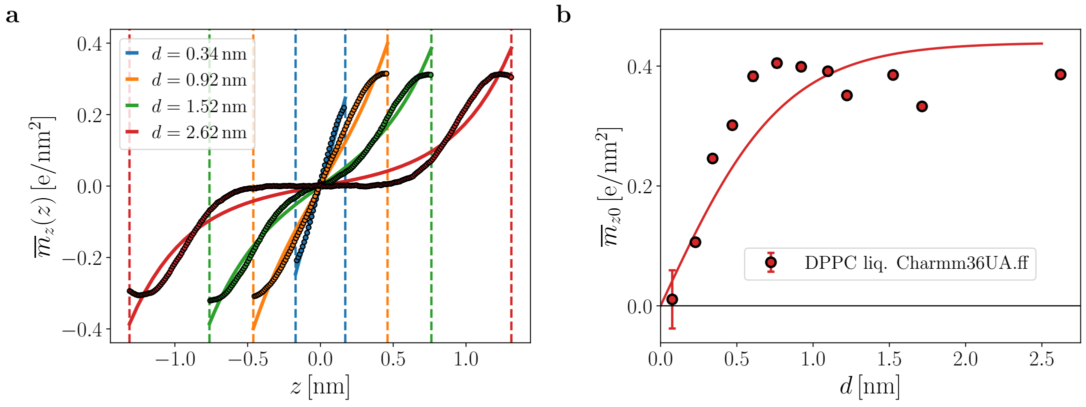

Figure S2. **Polarization order parameter for liquid DPPC using the C36-UA simulation parameters.** (a) Polarization profiles at different surface separations. (b) Order parameter at the surface (symbols) and fit of Eq. (6) of the main text to the simulation data (line). Data is obtained from simulations in the ensemble at fixed water chemical potential given by the bulk value at standard conditions.

Ginzburg prediction is excellent, although the deviations close to the interface positions are more pronounced compared to the Berger force-field results, which might be due to the complex atomistic head group derails in the case of C36-UA. However, the surface value of the polarization shown in Fig. S2 (b) is well described by Eq. (6) of the main text, corresponding to a constant surface-field boundary condition.

III. NON-LOCAL DENSITY-FUNCTIONAL THEORY FOR POLARIZABLE FLUIDS

Here we work out a few important properties of the non-local density-functional theory for polarizable fluids that we need in order to connect the scalar order parameter that appears in Eq. (1) of the main text with the normal component of the dielectric polarization. We write the electrostatic energy of the polarization-charge distribution $\rho_{\mathrm{pol}}(\mathbf{r})$ of a polarizable fluid using the bare Coulomb interaction $v(\mathbf{r}) = 1/(4\pi\varepsilon_0|\mathbf{r}|)$ as

$$\begin{aligned}\mathcal{H}_{\mathrm{pol}} =& \frac{1}{2}\int \mathrm{d}^3r\mathrm{d}^3r'\rho_{\mathrm{pol}}(\mathbf{r})v(\mathbf{r}-\mathbf{r}')\rho_{\mathrm{pol}}(\mathbf{r}') \\ =& \frac{1}{2}\int \mathrm{d}^3r\mathrm{d}^3r'\nabla_i m_i(\mathbf{r})v(\mathbf{r}-\mathbf{r}')\nabla_j m_j(\mathbf{r}') \\ =& \frac{1}{2}\int \frac{\mathrm{d}^3q}{(2\pi)^3}\tilde{m}_i(\mathbf{q})\tilde{m}_j(-\mathbf{q})q_iq_j/(\varepsilon_0 q^2).\end{aligned} \tag{S1}$$

Note that image-charge or dielectric-boundary effects do not modify the Coulomb interaction between polarization charges since polarization effects are explicitly accounted for. Throughout this derivation, we employ the Einstein convention, according to which doubly appearing indices are summed over, and define Fourier transforms as $\tilde{m}_i(\mathbf{q}) = \int \mathrm{d}^3r e^{-i\mathbf{q}\cdot\mathbf{r}}m_i(\mathbf{r})$. In the derivation of Eq. (S1) we used the relationship between the polarization $\mathbf{m}(\mathbf{r})$ and the polarization charge distribution, as described by the Poisson equation $\rho_{\mathrm{pol}}(\mathbf{r}) = -\nabla_i m_i(\mathbf{r})$ and inserted the Fourier transform of the Coulomb interaction $\tilde{v}(\mathbf{q}) = 1/(\varepsilon_0 q^2)$. The determinant of the matrix q_iq_j is zero; this signals a singularity of a purely dipolar interaction model that can be regularized by addition of an isotropic, wave-vector-dependent non-dipolar interaction $\delta_{ij}\tilde{f}(q^2)$. This non-dipolar interaction on one hand regulates the variance of the polarization, which reflects the fact that the molecular dipole moment is finite, on the other hand it accounts for the presence of non-electrostatic interactions. The resulting interaction is a free energy, since $\tilde{f}(q^2)$ will in general be temperature dependent, and can be written as

$$\mathcal{F}_{\mathrm{pol}} = \frac{1}{2}\int \frac{\mathrm{d}^3q}{(2\pi)^3}\left[\tilde{m}_i(\mathbf{q})\tilde{m}_j(-\mathbf{q})\frac{\tilde{g}^{-1}_{ij}(\mathbf{q})}{2} - \tilde{m}_i(\mathbf{q})\tilde{h}_i(-\mathbf{q})\right], \tag{S2}$$

where the interaction kernel is given by

$$\tilde{g}^{-1}_{ij}(\mathbf{q}) = \delta_{ij}\ \tilde{f}(q^2) + \frac{q_iq_j}{\varepsilon_0 q^2} \tag{S3}$$

and we have added a generating field $\tilde{h}_i(-\mathbf{q})$ that will be used to calculate expectation values further below. The matrix inverse of the interaction kernel is straightforwardly calculated as

$$\tilde{g}_{ij}(\mathbf{q}) = \frac{\delta_{ij}}{\tilde{f}(q^2)} - \frac{q_i q_j}{q^2\ \tilde{f}(q^2)\left[1+\varepsilon_0\ \tilde{f}(q^2)\right]}, \tag{S4}$$

the meaning of which will be interpreted further below. From Eq. (S4) it immediately transpires that the inverse of $\tilde{g}_{ij}(\mathbf{q})$ only exists in the presence of a finite non-dipolar interaction $\tilde{f}(q^2)$. All expectation values follow from the partition function $Z_{\rm pol} = \int \mathcal{D}\mathbf{m}(\cdot)\exp(-\beta\mathcal{F}_{\rm pol})$, the logarithm of which is, by completing the square, in real space given by $\ln(Z_{\rm pol}) = \beta\int \mathrm{d}^3r\mathrm{d}^3r' h_i(\mathbf{r})g_{ij}(\mathbf{r}-\mathbf{r}')h_j(\mathbf{r}')/2$. The mean polarization follows by taking a functional derivative as

$$\langle m_i(\mathbf{r})\rangle = \frac{\delta \ln Z_{\rm pol}}{\beta\delta h_i(\mathbf{r})} = \int \mathrm{d}^3r' g_{ij}(\mathbf{r}-\mathbf{r}')h_j(\mathbf{r}'), \tag{S5}$$

by which $g_{ij}(\mathbf{r}-\mathbf{r}')$ is recognized as the dielectric response function. In Fourier space it can be straightforwardly split into its longitudinal and transverse parts according to $\tilde{g}_{ij}(\mathbf{q}) = \tilde{g}^{\rm L}_{ij}(\mathbf{q}) + \tilde{g}^{\rm T}_{ij}(\mathbf{q})$ and which are defined by the properties $\tilde{g}^{\rm L}_{kj}(\mathbf{q}) = q_k q_i \tilde{g}_{ij}(\mathbf{q})$ and $q_i\tilde{g}^{\rm T}_{ij}(\mathbf{q}) = 0$. They are explicitly given by

$$\tilde{g}^{\rm L}_{ij}(\mathbf{q}) = \frac{\varepsilon_0 q_i q_j}{q^2\left[1+\varepsilon_0\ \tilde{f}(q^2)\right]}, \tag{S6}$$

$$\tilde{g}^{\rm T}_{ij}(\mathbf{q}) = \frac{\delta_{ij}\ - q_i q_j/q^2}{\tilde{f}(q^2)}. \tag{S7}$$

The polarization cumulant correlation function follows as

$$\begin{aligned} C_{ij}(\mathbf{r}-\mathbf{r}') = \langle m_i(\mathbf{r})m_j(\mathbf{r}')\rangle_C &= \frac{\delta^2 \ln(Z)}{\beta^2\delta h_i(\mathbf{r})\delta h_j(\mathbf{r}')} \\ &= \beta^{-1} g_{ij}(\mathbf{r}-\mathbf{r}'). \end{aligned} \tag{S8}$$

To obtain an expression for the bulk dielectric constant, we need to integrate $C_{ij}(\mathbf{r})$ over three-dimensional space, which is subtle. To highlight the salient properties of polarization correlations, we integrate $C_{ij}(\mathbf{r})$ over the xy-plane for polarizations that are perpendicular and parallel to that plane and obtain

$$\begin{aligned} \tilde{C}_\perp(q_z) &= \lim_{q_x,q_y\to 0}\tilde{C}_{zz}(\mathbf{q}) = \lim_{q_x,q_y\to 0}\tilde{g}_{zz}(\mathbf{q})/\beta \\ &= \lim_{q_x,q_y\to 0}\tilde{g}^{\rm L}_{zz}(\mathbf{q})/\beta = \frac{1}{\beta\left(\tilde{f}(q_z^2)+\varepsilon_0^{-1}\right)}, \end{aligned} \tag{S9}$$

$$\begin{aligned} \tilde{C}_\parallel(q_z) &= \lim_{q_x,q_y\to 0}\tilde{C}_{xx}(\mathbf{q}) = \lim_{q_x,q_y\to 0}\tilde{g}_{xx}(\mathbf{q})/\beta \\ &= \lim_{q_x,q_y\to 0}\tilde{g}^{\rm T}_{xx}(\mathbf{q})/\beta = \frac{1}{\beta\tilde{f}(q_z^2)}. \end{aligned} \tag{S10}$$

The two results are clearly different. We furthermore find that the perpendicular polarization correlations are solely determined by the longitudinal susceptibility, while the parallel correlations are solely determined by the transverse susceptibility. It transpires that the limit $\mathbf{q}\to\mathbf{0}$ depends on the order in which the components of $\mathbf{q}$ are sent to zero. The isotropic polarization correlation follows from Eqs. (S9) and (S10) as

$$\tilde{C}_{\rm iso}(q_z) = \tilde{C}_\perp(q_z) + 2\tilde{C}_\parallel(q_z) = \frac{2+3\varepsilon_0\tilde{f}(q_z^2)}{\beta\tilde{f}(q_z^2)\left(\varepsilon_0\tilde{f}(q_z^2)+1\right)}. \tag{S11}$$

Now the wave vector component q_z can be sent to zero without complications to obtain the total polarization variance as

$$\frac{\langle \mathbf{M}^2\rangle_C}{V} = \lim_{q_z\to 0}\tilde{C}_{\rm iso}(q_z) = \frac{2+3\varepsilon_0\tilde{f}_0}{\beta\tilde{f}_0\left(\varepsilon_0\tilde{f}_0+1\right)}, \tag{S12}$$

where we have introduced the limiting expression $\tilde{f}_0 = \tilde{f}(q^2 \to 0)$. According to the Kirkwood-Fröhlich formula [31], the relative dielectric constant ε of an infinite system (as used in the present calculation but not in the simulations, as is important to keep in mind) is related to the bulk polarization variance by

$$\frac{\beta\langle \mathbf{M}^2\rangle_C}{3\varepsilon_0 V} = \frac{(2\varepsilon+1)(\varepsilon-1)}{3\varepsilon}. \tag{S13}$$

Comparing Eqs. (S12) and (S13), we obtain the relative dielectric constant as

$$\varepsilon = 1 + \frac{1}{\varepsilon_0 \tilde{f}_0}. \tag{S14}$$

Using the result in Eq. (S14), the perpendicular and parallel correlations Eqs. (S9) and (S10) satisfy in the zero wave-vector limit the relations

$$\begin{aligned} \lim_{q_z\to 0} \frac{\beta \tilde{C}_\perp(q_z)}{\varepsilon_0} &= 1 - 1/\varepsilon, \\ \lim_{q_z\to 0} \frac{\beta \tilde{C}_\parallel(q_z)}{\varepsilon_0} &= \varepsilon - 1 \end{aligned} \tag{S15}$$

and thus exhibit a subtle symmetry breaking, which again reflects that the zero-wave vector limit of the polarization correlation function, $\tilde{C}_{ij}(\mathbf{q} \to \mathbf{0})$, for infinite systems depends on the order in which that limit is taken. The relations Eq. (S15) superficially resemble expressions used previously to extract perpendicular and parallel dielectric profiles from simulations of inhomogeneous systems in planar confinement with periodic boundary conditions and metallic embedding [30, 32]. In particular, the ratio of the parallel and perpendicular polarization fluctuations follows from Eq. (S15) as

$$\lim_{q_z\to 0} \frac{\tilde{C}_\parallel(q_z)}{\tilde{C}_\perp(q_z)} = \varepsilon, \tag{S16}$$

which shows that perpendicular polarization fluctuations are suppressed in comparison with parallel ones, in particular in water where $\varepsilon \approx 80$ [30, 32]. Note that this is not a confinement or boundary effect (there are no boundaries in the present calculations), but rather a dipolar tensorial effect that is also present in bulk systems.

In order to associate the generating field $h(\mathbf{r})$ with the electric field, we use the non-local linear response relation Eq. (S5). In Fourier space, and using the result in Eq. (S4), we obtain for the perpendicular and parallel response in the limit $q_x, q_y \to 0$

$$\begin{aligned} \frac{\langle \tilde{m}_z(q_z)\rangle}{\tilde{h}_z(q_z)} &= \frac{\varepsilon_0}{\varepsilon_0 \tilde{f}(q_z^2) + 1}, \\ \frac{\langle \tilde{m}_x(q_z)\rangle}{\tilde{h}_x(q_z)} &= \frac{1}{\tilde{f}(q_z^2)}. \end{aligned} \tag{S17}$$

The non-local linear dielectric response relation between the displacement field $\mathbf{D}(\mathbf{r})$ and the electric field $\mathbf{E}(\mathbf{r})$ reads

$$D_i(\mathbf{r}) = \varepsilon_0 \int \mathrm{d}^3 r' \varepsilon^{\mathrm{nl}}_{ij}(\mathbf{r}-\mathbf{r}') E_j(\mathbf{r}'), \tag{S18}$$

which defines the tensorial non-local dielectric function $\varepsilon^{\mathrm{nl}}_{ij}(\mathbf{r})$. Using the relation $\varepsilon_0 \mathbf{E}(\mathbf{r}) = \mathbf{D}(\mathbf{r}) - \mathbf{m}(\mathbf{r})$, we obtain in Fourier space in the limit $q_x, q_y \to 0$ the relations

$$\begin{aligned} \frac{\langle \tilde{m}_z(q_z)\rangle}{\tilde{D}_z(q_z)/\varepsilon_0} &= \varepsilon_0 \left(\tilde{\varepsilon}^{\mathrm{nl}}_{zz}(q_z) - 1\right), \\ \frac{\langle \tilde{m}_x(q_z)\rangle}{\tilde{E}_x(q_z)} &= \varepsilon_0 \left(1 - 1/\tilde{\varepsilon}^{\mathrm{nl}}_{xx}(q_z)\right). \end{aligned} \tag{S19}$$

Comparison with Eq. (S17) shows that the wave-vector dependent dielectric function is given by

$$\tilde{\varepsilon}^{\mathrm{nl}}_{zz}(q_z) = \tilde{\varepsilon}^{\mathrm{nl}}_{xx}(q_z) = 1 + \frac{1}{\varepsilon_0 \tilde{f}(q_z^2)} \tag{S20}$$

and that $\tilde{h}_z(q_z) = \tilde{D}_z(q_z)/\varepsilon_0$ and $\tilde{h}_x(q_z) = \tilde{E}_x(q_z)$. It thus turns out that the generating field h corresponds to the electric field E or to the displacement field D, depending on the direction. In the limit $q_z \to 0$ the wave-vector dependent dielectric function Eq. (S20) goes over to the bulk dielectric constant Eq. (S14). In this respect it is important to note that in the time-independent case, E_x is independent of z, while in the absence of free charges, D_z is independent of z. So in a simulation, one determines the wave-vector dependent dielectric response from the polarization correlation function Eq. (S9) [33].

We finally note that in an isotropic system, there are two equivalent ways of calculating the isotropic correlation function $C_{\text{iso}}(r)$, defined in Eq. (S11), in real space, either by taking the trace of the tensorial polarization correlation function,

$$C_{\text{iso}}(r) = C_{xx}(\mathbf{r}) + C_{yy}(\mathbf{r}) + C_{zz}(\mathbf{r}), \tag{S21}$$

or by an angular average over one diagonal component of the tensorial polarization correlation function,

$$C_{\text{iso}}(r) = 3 \int \frac{\mathrm{d}\Omega}{4\pi} C_{zz}(\mathbf{r}). \tag{S22}$$

IV. MAPPING BETWEEN LANDAU–GINZBURG MODEL AND NON-LOCAL POLARIZATION THEORY

In the following, we show how to relate the parameters a and b of the one-dimensional Landau–Ginzburg model in Eq. (1) of the main text to the parameters appearing in the augmented polarization free energy defined by Eqs. (S2) and (S3). We consider the polarization in z-direction, $m_z(x, y, z)$, that is averaged over the lateral x and y directions, so the relevant interaction kernel is given by

$$\tilde{g}_{zz}^{-1}(q_z) = \lim_{q_x, q_y \to 0} \tilde{g}_{zz}^{-1}(\mathbf{q}) = \frac{1}{\varepsilon_0} + \tilde{f}(q_z^2), \tag{S23}$$

which after expansion of the function $\tilde{f}(q_z^2)$ to first order according to $\tilde{f}(q_z^2) \simeq \tilde{f}_0 + q_z^2 \tilde{f}_1$ results in

$$\tilde{g}_{zz}^{-1}(q_z) = \frac{1}{\varepsilon_0} + \tilde{f}_0 + \tilde{f}_1 q_z^2. \tag{S24}$$

Using the kernel in Eq. (S24) in conjunction with Eq. (S2) and comparing with Eq. (1), we obtain the parameters as

$$\begin{aligned} a &= \frac{\beta}{2}\left(\frac{1}{\varepsilon_0} + \tilde{f}_0\right) = \frac{1}{2\tilde{C}_\perp(q_z = 0)} = \frac{\beta\varepsilon}{2\varepsilon_0(\varepsilon - 1)} \\ b &= \frac{\beta f_1}{2}, \end{aligned} \tag{S25}$$

where in the last expression for a we have used the result in Eq. (S14) for the relative bulk dielectric constant ε.

From Eq. (S25) we obtain $a = 355\,\text{nm}/\text{e}^2$, where we used $\varepsilon_{\text{bulk}} = 70$ for SPC/E water at 300 K [30, 34]. Note, however, that Eq. (S14) only holds for a homogeneous bulk system; interfacial and confined systems are characterized by anisotropic polarization fluctuations, where the perpendicular polarization fluctuations are significantly suppressed for confined water while the perpendicular dielectric constant stays rather constant and decreases only for strong confinement of about a nanometer [30, 35, 36]. For an interfacial system we can estimate a from the perpendicular polarization fluctuations. Using that $\tilde{C}_\perp(q_z = 0) = \langle M_z^2 \rangle_C / V$ we obtain from Eq. (S25) the relation

$$a = \frac{1}{2\langle M_z^2 \rangle_C / V}. \tag{S26}$$

V. LANDAU FREE ENERGY WITH GENERALIZED BOUNDARY CONDITIONS

We derive the solution of the Landau–Ginzburg model including surface fields h and g that couple linearly and quadratically to the order parameter $\overline{m}_z$ [37, 38]. The free energy in quadratic order is given by

$$\frac{\beta\mathcal{F}_{\rm LG}\left[\overline{m}_z(\cdot)\right]}{A} = \int_{-d/2}^{d/2}\left[a\overline{m}_z^2(z) + b\left(\nabla\overline{m}_z(z)\right)^2\right]{\rm d}z + h_+\overline{m}_z\left(\frac{d}{2}\right) + h_-\overline{m}_z\left(-\frac{d}{2}\right) + g_+\overline{m}_z^2\left(\frac{d}{2}\right) + g_-\overline{m}_z^2\left(-\frac{d}{2}\right). \quad \text{(S27)}$$

Performing the variation yields

$$\begin{aligned}\frac{\beta\delta\mathcal{F}_{\rm LG}[\overline{m}_z(\cdot)]/A}{\delta\overline{m}_z(\tilde{z})} &= 2a\overline{m}_z(\tilde{z}) - 2b\nabla^2\overline{m}_z(\tilde{z}) + \delta\left(\frac{d}{2}-\tilde{z}\right)\left[h_+ + 2b\nabla\overline{m}_z\left(\frac{d}{2}\right) + 2g_+\overline{m}_z\left(\frac{d}{2}\right)\right] \\ &\quad + \delta\left(\frac{d}{2}+\tilde{z}\right)\left[h_- - 2b\nabla\left(-\frac{d}{2}\right) + 2g_-\overline{m}_z\left(-\frac{d}{2}\right)\right]. \end{aligned} \quad \text{(S28)}$$

The antisymmetric solution for $h_+ = -h_- \equiv h$ and $g_+ = +g_- \equiv g$ of Eq. (S28) is given by [38]

$$\overline{m}_z(z) = -\frac{h\lambda}{2b}\frac{\sinh\left(z/\lambda\right)}{\cosh(d/2\lambda) + \chi\sinh\left(d/2\lambda\right)}, \quad \text{(S29)}$$

where we have used $\lambda = (b/a)^{1/2}$ and $\chi = g\lambda/b = g(ab)^{-1/2}$. The free energy then follows from Eq. (S27) as

$$\frac{\beta\mathcal{F}_{\rm LG}}{A} = \frac{h^2\lambda}{2b}\frac{1}{\chi + \coth(d/2\lambda)}. \quad \text{(S30)}$$

For $\chi \to 0$, Eq. (5) of the main text is recovered and the surface value $\overline{m}_{z0} = \pm\overline{m}_z(\pm d/2)$ follows from the constant coupling field h,

$$\overline{m}_{z0} = \frac{h}{2a\lambda}\tanh(d/2\lambda). \quad \text{(S31)}$$

In this case, the free energy simplifies to

$$\frac{\beta\mathcal{F}_{\rm LG}}{A} = \frac{h^2\lambda}{2b}\frac{1}{\coth(d/2\lambda)}, \quad \text{(S32)}$$

as obtained by Cevc et al. [39]. We used the limiting case $\chi \to 0$ for our analysis in the main text because the simulation results can be modeled rather well using Eq. (S31). The resulting pressure is repulsive and follows from the derivative of Eq. (S32) as

$$\Pi_{\rm ind} = -\frac{\partial}{\partial d}\frac{\beta\mathcal{F}_{\rm LG}}{A} = \frac{h^2}{4b}\frac{1}{\cosh^2(d/2\lambda)}, \quad \text{(S33)}$$

where it is noted that the Landau–Ginzburg model does not account for direct membrane–membrane interactions and thus only describes the indirect contribution to the pressure.

In the opposite limit for large χ, the surface stiffness g dominates. Expanding Eq. (S30) to second order results in

$$\frac{\beta\mathcal{F}_{\rm LG}}{A} \simeq -\frac{h^2\lambda}{2b}\left[\frac{1}{\chi} - \frac{\coth\left(d/2\lambda\right)}{\chi^2}\right] \quad \text{(S34)}$$

and the corresponding pressure follows as

$$\Pi_{\rm ind} = -\frac{\partial}{\partial d}\frac{\beta\mathcal{F}_{\rm LG}}{A} \simeq -a\overline{m}_{z0}^2\frac{1}{\sinh^2(d/2\lambda)}, \quad \text{(S35)}$$

which reveals attraction. Note that for $d \gg \lambda$ both expressions Eqs. (S33) and (S35) show an exponential decay,

$$\Pi_{\rm ind}(d \gg \lambda) \sim \pm{\rm e}^{-d/\lambda}, \quad \text{(S36)}$$

however, the behavior at small separations is very different [40]: Whereas for $\chi \to 0$ the pressure saturates for fixed $\overline{m}_{z0}$, it diverges for $\chi \to \infty$.

The solution for symmetric order parameters $h_+ = h_- \equiv h$ and $g_+ = g_- \equiv g$ follows as

$$\overline{m}_z(z) = -\frac{h\lambda}{2b}\frac{\cosh\left(z/\lambda\right)}{\cosh(d/2\lambda) + \chi\sinh\left(d/2\lambda\right)} \quad \text{(S37)}$$

with the free energy

$$\frac{\beta\mathcal{F}_{\rm LG}}{A} = -\frac{h^2\lambda}{2b}\frac{1}{\chi + \tanh(d/2\lambda)}. \tag{S38}$$

In the limit $\chi \to 0$ the profile simplifies to

$$\overline{m}_z(z) = \overline{m}_{z\,0}\frac{\cosh\left(z/\lambda\right)}{\cosh(d/2\lambda)}, \tag{S39}$$

where the surface value of the order parameter is given by [40]

$$\overline{m}_{z0} = \frac{h}{2a\lambda}\coth(d/2\lambda) \tag{S40}$$

and the resulting interaction pressure,

$$\Pi_{\rm ind} = -\frac{\partial}{\partial d}\frac{\beta\mathcal{F}_{\rm LG}}{A} = -\frac{h^2}{4b}\frac{1}{\sinh^2(d/2\lambda)}, \tag{S41}$$

is attractive.

In the limit of large χ the free energy follows to second order as

$$\frac{\beta\mathcal{F}_{\rm LG}}{A} \simeq -\frac{h^2\lambda}{2b}\left[\frac{1}{\chi} - \frac{\tanh\left(d/2\lambda\right)}{\chi^2}\right] \tag{S42}$$

and the pressure as

$$\Pi_{\rm ind} = -\frac{\partial}{\partial d}\frac{\beta\mathcal{F}_{\rm LG}}{A} = a\overline{m}_{z0}^2\frac{1}{\cosh^2(d/2\lambda)}. \tag{S43}$$

Thus, when $\overline{m}_z(\pm d/2) = \overline{m}_{z0}$ is constant, the pressure is repulsive.

VI. QUADRUPOLE AND OCTUPOLE ORDER-PARAMETER PROFILES

There are many order parameters that can be used to describe the water structure, here we discuss higher-order electric multipole densities.

A. Octupole order parameter

We start with the zzz-component of the octupole density, $\overline{o}_{zzz}(z) = \left\langle \overline{O}_{zzz}(z)\rho(z)\right\rangle$, where, as defined in the main text, the overline denotes the lateral average. Because of its symmetry, the zzz-component of the octupole density is expected to exhibit an antisymmetric profile between identical surfaces, therefore it will make a repulsive contribution to the total hydration pressure for the constant surface-field boundary condition. The octupole moment of molecule i is defined in terms of the multipole expansion with $l = 3$ as $O_{zzz} = (1/6)\sum_{j(i)} q_j(z_j - z_i)^3$, where as reference position of a water molecule we chose the oxygen atom. Figure S3 shows the profiles $\overline{o}_{zzz}(z)$ for decanol and DPPC in the liquid and gel phase, together with the fits to Eq. (5) of the main text. The resulting values for the decay lengths are $\lambda_{\overline{o}} = 0.15\,\text{nm}$ for decanol and 0.30 and 0.28 nm for DPPC in the liquid and gel phase, respectively, and are close to the values given for the polarization in table 1 of the main text. The surface values shown in Fig. S4 are also nicely described by the Landau–Ginzburg prediction for constant surface-field boundary condition, Eq. (6) of the main text.

To obtain the expected amplitude of the indirect pressures due to the octupole ordering $\beta\Pi^{\infty}_{\text{ind},\overline{o}} = h^2_{\overline{o}}/\left(a_{\overline{o}}\lambda^2_{\overline{o}}\right)$ as defined in Eq. (8) of the main text, we need to independently determine the corresponding stiffness $a_{\overline{o}}$, note that $\lambda_{\overline{o}}$ is already determined from the fits in Fig. S3 and the ratio $h_{\overline{o}}/a_{\overline{o}}$ follows from the fits in Fig. S4 according to Eq. (6) of the main text. For an interfacial system the expression corresponding to Eq. (S26) for the octupolar order parameter is

$$a_{\overline{o}} = \frac{1}{2\langle\overline{O}^2_{zzz}\rangle/V}, \tag{S44}$$

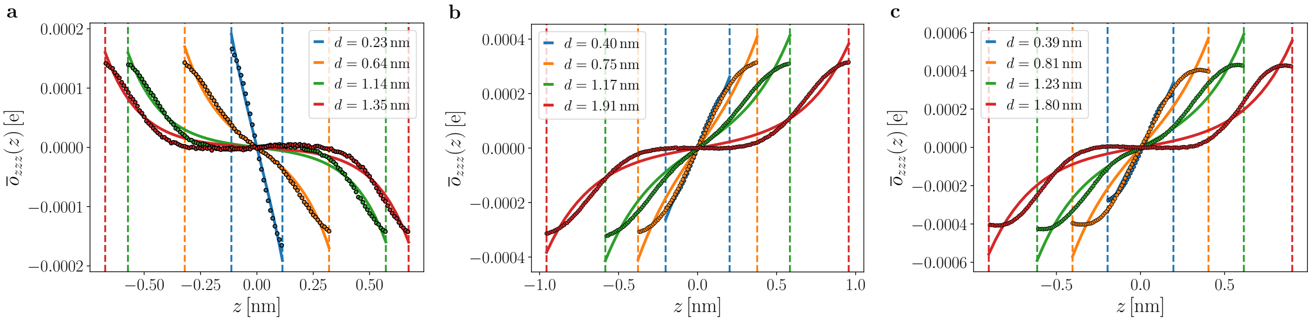

Figure S3. **Octupole order-parameter profiles.** Simulation data for (a) decanol bilayers, (b) DPPC bilayers in the liquid L_α phase and (c) DPPC bilayers in the gel L_β phase. The dashed vertical lines indicate the surface positions at $\pm d/2$, the corresponding values of d are given in the legends. Solid lines are fits according to Eq. (5) given in the main text, yielding decay lengths $\lambda_{\bar{o}} = 0.15$, 0.30 and 0.28 nm, respectively, for (a)-(c). Data is obtained from simulations in the constant water chemical potential ensemble.

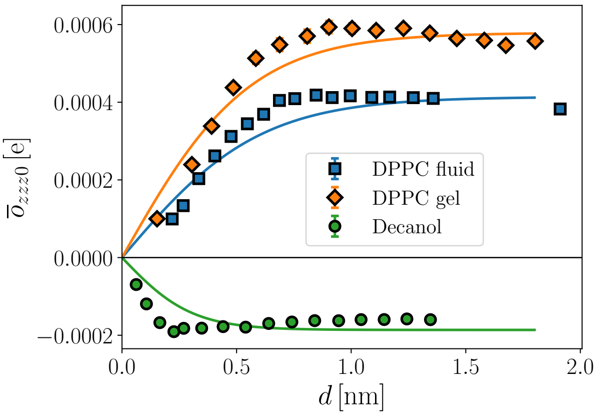

Figure S4. **Octupole order-parameter surface values:** Data is shown for decanol (green), DPPC in the liquid state (blue) and DPPC in the gel phase (orange). Lines denote fits to Eq. (6) given in the main text where the amplitudes h/a are fitted to the simulation data while the corresponding correlation lengths $\lambda_{\bar{o}}$ are taken from the fits in Fig. S3.

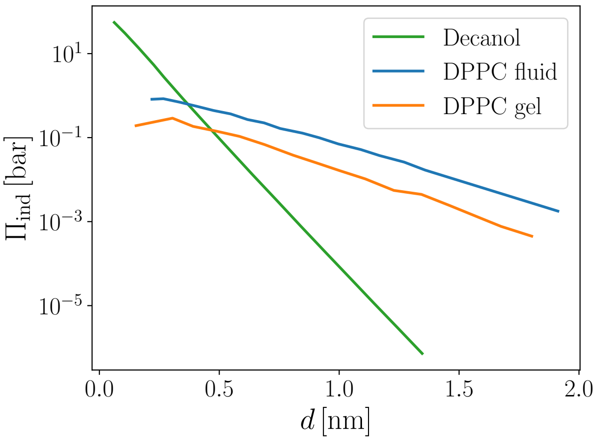

Figure S5. **Landau–Ginzburg pressure due to water octupole ordering.** The predicted repulsive indirect pressure due to the distance-dependent perturbation of the water octupole order-parameter profile is obtained from the fits in Figs. S3 and S4 and the stiffness calculated according to Eq. (S44). As before, data is shown for decanol and DPPC in the L_α and L_β phases, respectively.

where $V = Ad$ is the volume corresponding to the surface separation d between the surfaces of area A. In our simulations we evaluate the instantaneous laterally averaged octupole moment of the confined water by summing over the octupole moments of water molecule i according to $\overline{O}_{zzz} = \int_{-L/2}^{L/2} \mathrm{d}z\, \overline{o}_{zzz}(z) = \sum_{i=1}^{N_\mathrm{w}} O_z zz^{(i)}$.

The repulsive indirect pressures predicted by the Landau–Ginzburg model for the octupolar order parameter are shown in Fig. S5. As expected from the good agreement of the order parameter profiles between Landau–Ginzburg predictions and simulations, the shape of the pressure–distance curve is quite similar to the indirect pressures from simulations reported in Fig. 3 of the main text, but the amplitudes are significantly smaller by a factor of about 10^2 (for decanol) and 10^4 (for DPPC), thus rendering the contribution of the one-dimensional, laterally averaged octupole density orientation to the indirect pressure negligible.

B. Quadrupole order parameter

As an example for a symmetric order parameter we discuss the laterally averaged water quadrupole density $\overline{q}_{zz}$. In analogy to the main text and the analysis of the octupole density discussed above, we show the quadrupole density $\overline{q}_{zz}(z) = \big\langle (\overline{Q}_{zz}(z) - Q_\mathrm{bulk})\rho(z) \big\rangle$ in Fig. S6, where we subtract the bulk value $Q_\mathrm{bulk} = 1.411 \cdot 10^{-3}\,\mathrm{e\,nm}^2$. The quadrupole density is even with respect to the $z = 0$ plane, as expected by symmetry. The quadrupole density profiles shown in Fig. S6 can be fitted rather well to the symmetric expression Eq. (S39) derived for constant surface-field boundary condition. The decay lengths obtained from these fits, $\lambda_{\overline{q}} = 0.08$, 0.22 and 0.20 nm for the decanol bilayer, DPPC in the fluid and DPPC in the gel phase, respectively, are similar to the values for the polarization given in table 1 of the main text.

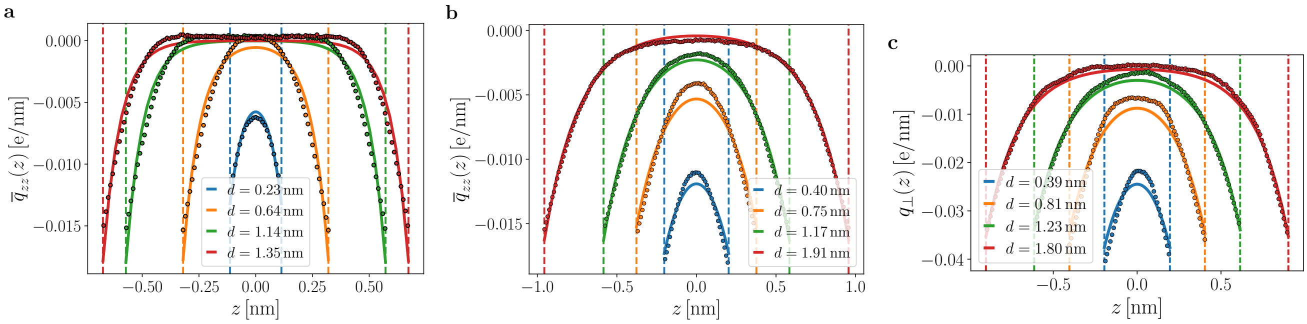

Figure S6. **Quadrupole order-parameter profiles.** Simulation data for (a) decanol bilayers, (b) DPPC bilayers in the liquid L_α phase and (c) DPPC bilayers in the gel L_β phase. The dashed vertical lines indicate the surface positions at $\pm d/2$, the corresponding values of d are given in the legends. Solid lines are fits according to Eq. (S39) yielding decay lengths $\lambda_{\overline{q}} = 0.08$, 0.22 and 0.20 nm, respectively, for (a)-(c). Data is obtained from simulations in the constant water chemical potential ensemble.

The surface values q_{zz0} obtained from the fits are shown in Fig. S7 and are satisfactorily described by Eq. (S40), thus confirming the constant surface-field boundary condition also for the quadrupolar density.

Using the same methodology as outline in the previous section, we evaluate the pressure due to the quadrupole ordering using the corresponding stiffness

$$a_{\overline{q}} = \frac{1}{2\langle \overline{Q}_{zz}^2 \rangle / V}. \tag{S45}$$

Since the constant surface field boundary condition holds also for the quadrupole order parameter, the resulting indirect pressures according to the Landau–Ginzburg model are attractive and shown in Fig. S8. The pressures for decanol and DPPC in the liquid and gel phase are not only attractive, but also two to three orders of magnitude smaller than the repulsion due to polarization in Fig. 3 of the main text and thus completely negligible.

Although there exist a large variety of other order parameters that can be used to describe the water structure, the most intuitive candidates, such as the second Legendre polynomial of the water orientation or the water tetrahedrality, are symmetric and obey the constant surface-field boundary condition [41], they thus lead to attraction and do not serve to explain the hydration repulsion.

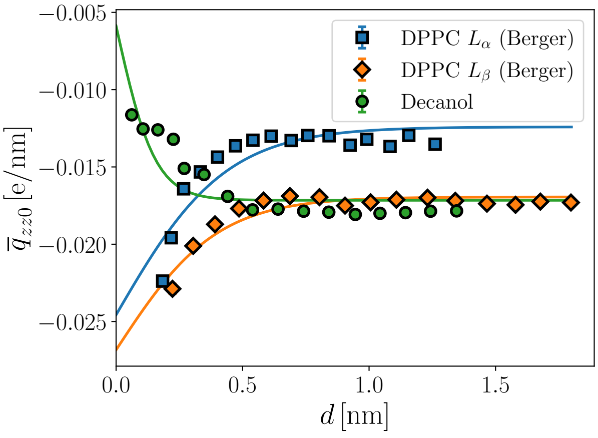

Figure S7. **Quadrupole order-parameter surface values:** Data is shown for decanol (green), DPPC in the liquid state (blue) and DPPC in the gel phase (orange). Lines denote fits of Eq. (S40) to the simulation data yielding the amplitudes h/a, while the corresponding correlation lengths $\lambda_{\bar{q}}$ are taken from the fits in Fig. S6.

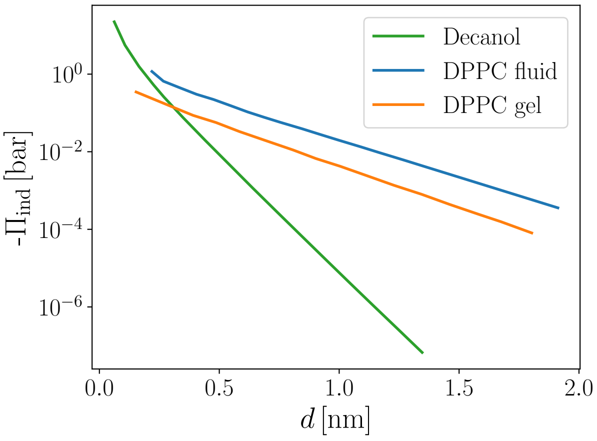

Figure S8. **Landau–Ginzburg pressure due to water quadrupole ordering.** The predicted attractive indirect pressure due to the distance-dependent perturbation of the quadrupolar order-parameter profile is obtained from the fits in Figs. S6 and S7 and the stiffness calculated according to Eq. (S45). As before, data is shown for decanol and DPPC in the L_α and L_β phases, respectively.

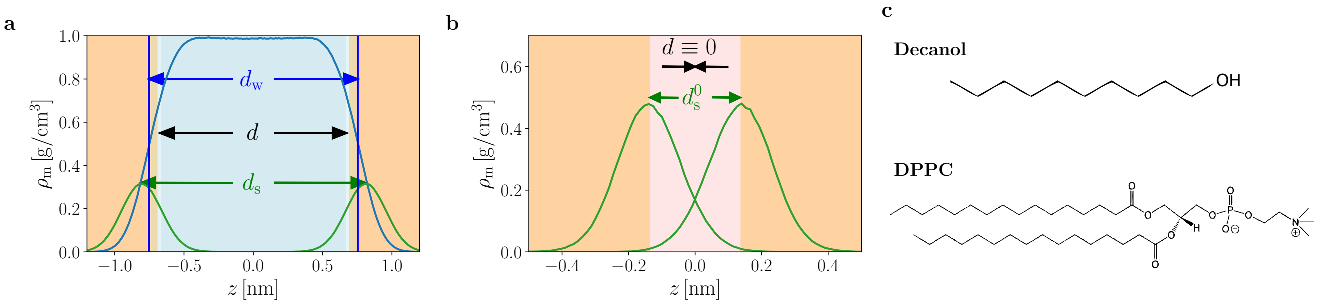

Figure S9. **Definition of the separation d.** (a) Mass density ρ_m of water (blue line) and the oxygen of the head group (green line) for the decanol system at a water slab thickness $d_\text{w} = 1.5\,\text{nm}$. The structural separation d_s is defined by the separation between the mean position of the selected head group atoms, for decanol the oxygens and for DPPC the posphors. Vertical blue lines indicate the Gibbs dividing surface positions, whose separation defines d_w, the light blue shaded area denotes the distance d, which follows from the difference between the structural separation d_s at finite water content and its value at zero water content $N_\text{w} = 0$ and extrapolated pressure, shown in (b). (c) Chemical structure of a decanol molecule and a DPPC lipid molecule.

VII. DISTANCE DEFINITION

In Fig. S9(a) we illustrate the determination of the distance d based on the structural separation d_s between selected atoms in the head group, for which we chose the oxygen for decanol and phosphor for DPPC. To define the head group positions we evaluate the center of mass of the selected atoms. The equilibrium separation at zero water content d_s^0 is determined from simulations at 1 bar normal pressure in the absence of water, $d_\mathrm{w} = 0$ (c.f. Fig. 3 of the main text), as illustrated in Fig. S9(b).

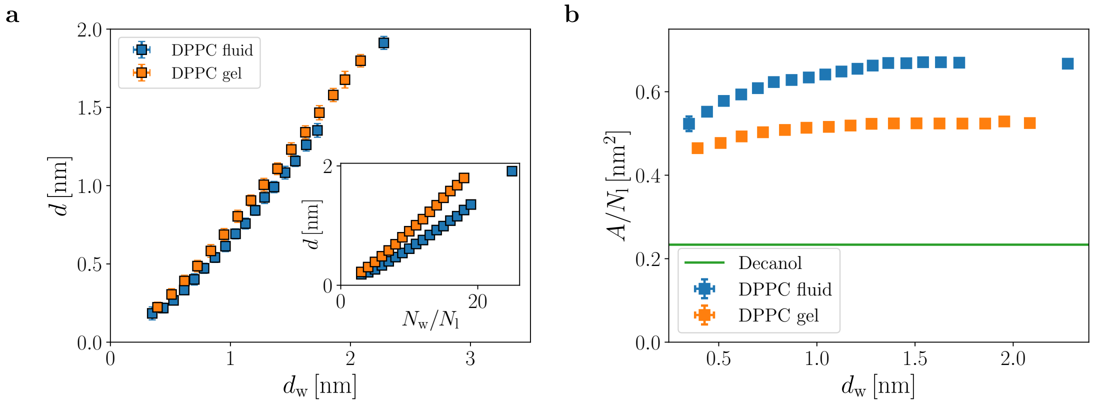

Figure S10. **Structural separation and lateral area.** (a) Separation d based on the phosphorus distance for DPPC bilayers as a function of the water slab thickness d_w in the osmotic stress ensemble. The inset shows d as a function of the number of waters per head group, $N_\mathrm{w}/N_\mathrm{l}$. (b) Lateral area A per lipid molecule N_l. For DPPC (symbols), the area is free to adjust during the dehydration, whereas for decanol the lateral area in the simulations is fixed.

Figure S10(a) shows a comparison of the effective distance d, defined based on the separation between selected atoms in the head group, versus the water slab thickness d_w, based on the bulk water molecular volume v_w according to $d_\mathrm{w} = N_\mathrm{w} v_\mathrm{w}/A$, for DPPC bilayer systems considered. We find for separations $d_\mathrm{w} \gtrsim 1\,\mathrm{nm}$ a roughly linear relation between d and d_w, which for smaller separations turns into a non-linear relation (note that by definition $d \to 0$ as $d_\mathrm{w} \to 0$). This becomes clear when considering the inset of Fig. S10(a), which shows d as a function of the number of waters per lipid molecule, $N_\mathrm{w}/N_\mathrm{l}$. The different slopes reflect the different areas per lipid molecule via $N_\mathrm{w}/N_\mathrm{l} = A d_\mathrm{w}/(v_\mathrm{w} N_\mathrm{l})$, shown in Fig. S10(b). Whereas for simulations of DPPC the lateral area adjusts such that the lateral pressure is 1 bar, which corresponds to the experimental situation in the osmotic stress ensemble [19], we fix the area in the simulations of decanol independently of the hydration.

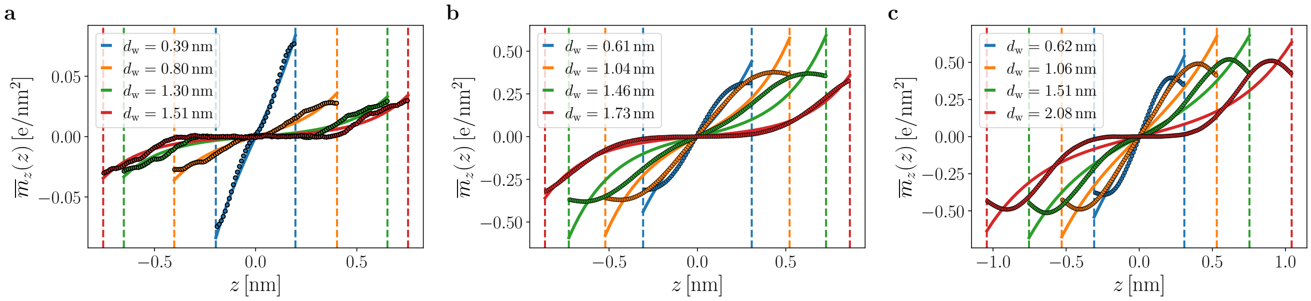

Figure S11. **Polarization density profiles using d_w as surface position definition.** (a) Decanol bilayers, (b) DPPC in the disordered L_α and (c) DPPC in the ordered L_β phase. Data is obtained from simulations in the constant water chemical potential ensemble.

To justify our definition of d we show in Fig. S11 the polarization profiles for the three systems where the water slab thickness d_w is used for the effective separation that enters the Landau–Ginzburg model. The corresponding surface values $m_{\perp 0}$ obtained from fitting Eq. (5) of the main text to the simulation data in Fig. S11 are shown in Fig. S12. The qualitative behavior is similar to the case when d is used for the effective distance in Figs. 4 and 5 in the main text, however, the quality of the fits is slightly worse.

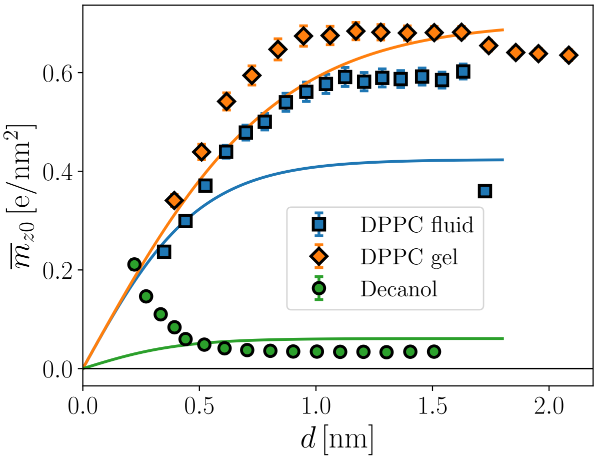

Figure S12. **Polarization order-parameter surface values using d_w as surface-position definition.** The values $m_{\perp 0}$ are obtained from the fits of the polarization profiles, shown in Fig. S11, to Eq. (5) of the main text.

VIII. THERMODYNAMIC EXTRAPOLATION METHOD

The Gibbs–Duhem equation for bulk water, $N_\mathrm{w}\,\mathrm{d}\mu = -S\mathrm{d}T + V\mathrm{d}\Pi$, evaluated at constant temperature yields

$$\left(\frac{\partial \mu}{\partial \Pi}\right)_T = \frac{V}{N_\mathrm{w}} = v_\mathrm{w}(\Pi). \tag{S46}$$

From Eq. (S46) the chemical potential difference between a water reservoir at chemical potential μ_osm and pressure Π_osm and water confined between two surfaces at (hydrostatic) interaction pressure Π follows as

$$\Delta\mu = \mu - \mu_\mathrm{osm} = \int_{\Pi_\mathrm{osm}}^{\Pi} v_\mathrm{w}(\Pi')\,\mathrm{d}\Pi'. \tag{S47}$$

In osmotic stress experiments, as well as in the corresponding simulations, the pressure is fixed by atmospheric conditions $\Pi_\mathrm{osm} \approx 1$ bar. Using the fact that water is incompressible up to several kilobars [8] allows us to approximate the relation using the constant bulk water volume v_w as

$$\Delta\Pi = \Pi - \Pi_\mathrm{osm} = \frac{\Delta\mu}{v_\mathrm{w}} + \mathcal{O}(\Delta\Pi^2). \tag{S48}$$

While the bulk water volume can be measured directly in simulations, the accurate determination of chemical potentials is more involved, see e.g. Ref. [42]. We decompose the chemical potential according to

$$\mu(z) = k_B T \log\rho(z) + \mu_\mathrm{LJ}(z) + \mu_\mathrm{C}(z), \tag{S49}$$

where the first term is the ideal gas contribution and $\rho(z)$ is the water-mass density at position z. The other two terms correspond to the excess Lennard-Jones (LJ) and Coulomb contributions, respectively. We make use of the fact that a SPC/E water molecule consists of a single LJ interaction site only, therefore it is convenient to evaluate μ_LJ via the Widom Test Particle Insertion method (TPI) [43]. The electrostatic chemical potential μ_C is then estimated using the MBAR method [9]. Note that in thermodynamic equilibrium, the total chemical potential μ is independent of the position z, therefore it can be evaluated at an arbitrary position, which we choose in the center of the water slab between the surfaces.

IX. DECOMPOSITION OF THE INTERACTION PRESSURE

In order to relate the interaction pressure from simulations to the Landau–Ginzburg model, we decompose the pressure into direct, i.e. bilayer-bilayer contributions, and an indirect part, which is mediated by water. To this end we post-process the simulation trajectories by expanding the simulation box in z-direction such that on each

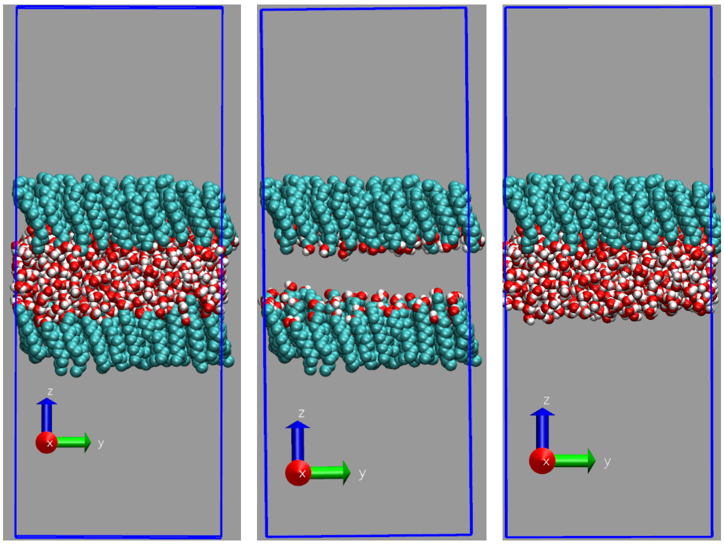

(a) (b) (c)

Figure S13. **Simulation setup for pressure decomposition into direct and indirect contributions, here illustrated for decanol bilayers.** (a) The total force acting on the upper monolayer is measured in a box that is expanded such that periodic replica can be neglected. (b) To obtain the direct pressure the water is removed and the force on the upper monolayer is measured. (c) To obtain the interaction pressure between water and the upper surface, the lower monolayer is removed.

side of the water slab, which is in the center of the box, there is only one monolayer, see Fig. S13 (a). This way interactions with periodic images in z-direction are minimized, as Lennard-Jones interactions are zero beyond the cut-off r_c and electrostatic interactions, which to leading order are due to dipolar interactions, decay also relatively quickly. We verified that the pseudo-2D summation of the electrostatic forces [44] does not change this decomposition, as the monolayers are net neutral and the negligible dipole term does not change the pressure. To measure the direct contribution, the water is removed from the slab, thus only the opposing monolayers, which interact with each other across the free space, see Fig. S13 (b), are left. The force acting on one of the monolayers divided by the area gives the direct contribution to the hydration pressure. In order to obtain the indirect contribution, one monolayer is removed, and again the force acting on the remaining monolayer is calculated, see Fig. S13 (c). The obtained sum of the pressures $\Pi = \Pi_{\mathrm{ind}} + \Pi_{\mathrm{dir}}$ determined from the force acting on the surfaces agrees perfectly with the values obtained from the virial (circles in Fig. 3(a) of the main text), demonstrating that interactions between periodic neighbors contribute negligibly to the hydration force; this validates our method used for the pressure decomposition.

X. MULTIPOLE EXPANSION OF THE POLARIZATION DENSITY AND FACTORIZED DENSITY-WEIGHTED DIPOLE DENSITY

In Figure S14(a) we show the water polarization density and its multipole contributions up to the octupole moment according to Eq. (10) of the main text for the decanol bilayer system at $d = 1.35\,\mathrm{nm}$ and in Fig. S14(b) for DPPC in the liquid state at $d = 1.72\,\mathrm{nm}$. In both cases the octupole term $\overline{m}_z^{(3)}$ (red line in Fig. S14(a) and (b)) is zero within the numerical noise. Smoothing the octupole data via a gliding window average before taking the derivative, shown in the inset of Fig. S14(a), does not yield profiles that agree well with the sinh-shape expected from the Landau–Ginzburg model in Eq. (5) of the main text. As discussed there, the quadrupole contribution for decanol over-compensates the dipole contribution, giving rise to different signs between $\overline{m}_z^{(1)}$ and $\overline{m}_z$. Contrary, for DPPC in Fig. S14(b) the quadrupole contribution has the same sign as the dipole contribution and contributes only about 10% to the total polarization.

It is worthy to note that the water density profile and the water dipolar orientation profile are broad distributions that do not factorize. This is demonstrated in Fig. S14(c), where we plot the dipolar polarization density profile $\overline{m}_z^{(1)}(z)$ (orange line), which in our analysis is computed as $\overline{m}_z^{(1)}(z) = P_0 \langle \rho(z) \cos\theta(z) \rangle$ from the average over the product of the water density ρ and its orientation with respect to the membrane normal $\cos(\theta)$, where $P_0 = 4.893 \cdot 10^{-2}\,\mathrm{e\,nm}$ is the dipole moment of SPC/E water. The factorized expression, given by $P_0 \langle \rho(z) \rangle \langle \cos\theta(z) \rangle$ (blue line), is slightly

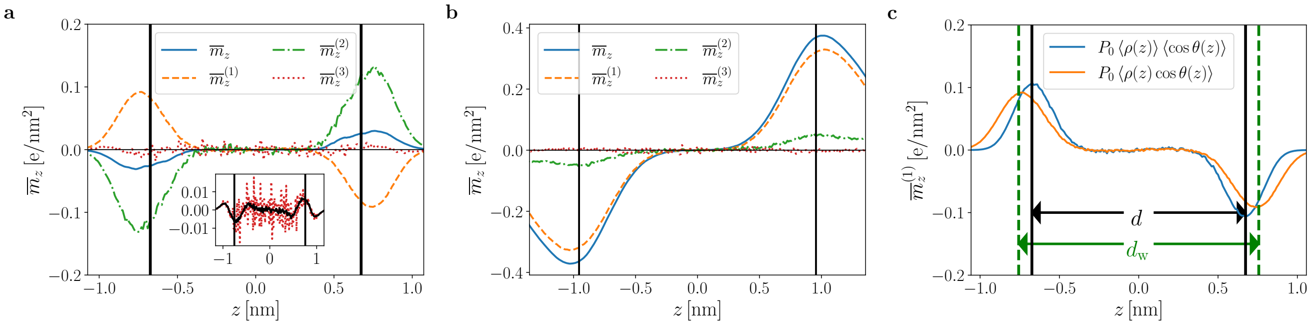

Figure S14. **Multipole expansion of the water polarization density.** (a) Polarization density for the decanol system at $d = 1.35\,\mathrm{nm}$. The total water polarization (blue) is split into contributions stemming from dipoles (orange), quadrupoles (green) and octupoles (red), we find the octupole term to be almost negligible and dominated by numerical noise. Vertical black lines indicate the surface positions defined by the separation d. The inset shows the smoothed contribution of the octupole moment density. (b) Same as in (a) but for DPPC in the liquid state at $d = 1.72\,\mathrm{nm}$. (c) Average dipole density (orange line) and density weighted dipole orientation (blue line) for decanol at $d = 1.35\,\mathrm{nm}$. The vertical black lines denote the corresponding interface positions defined by the separation d, dashed green lines the position of the GDS corresponding to $d_{\mathrm{w}} = 1.5\,\mathrm{nm}$.

shifted, indicating that the distributions of density and orientation do not factorize. Interestingly, the product $P_0 \langle \rho(z) \rangle \langle \cos\Theta(z) \rangle$ has its maximum at the position of the surface defined by the shifted structural distance d, indicated as vertical solid black lines in Fig. S14(c).

XI. LATERALLY INHOMOGENEOUS ORDER-PARAMETER PROFILES

We so far have considered the Landau–Ginzburg model only for the laterally averaged polarization, which due to isotropy corresponds to the polarization in z-direction $\overline{m}_z(z)$. We here consider a more general vectorial polarization profile

$$m_i(\vec{r}) = m_i^q(z)\cos(\bar{q}_x x) + \overline{m}_z(z) \tag{S50}$$

with $i = x, y, z$, that exhibits a modulation in x-direction characterized by the wave vector $\bar{q}_x$. The z-dependent coefficients $m_i^q(z)$ describe the modulated components of the polarization profile. The Fourier-transformed polarization profile reads

$$\begin{aligned}\tilde{m}_i(\vec{q}) = {}& \tilde{m}_i^q(q_z)2\pi\delta(q_y)\pi\left[\delta(q_x - \bar{q}_x) + \delta(q_x + \bar{q}_x)\right] \\ & + \tilde{\overline{m}}_z(q_z)2\pi\delta(q_x)2\pi\delta(q_y).\end{aligned} \tag{S51}$$

In analogy to Eq. (S2), the polarization free energy is given as

$$\mathcal{F}_{\mathrm{pol}} = \frac{1}{2}\int \frac{\mathrm{d}^3 q}{(2\pi)^3}\tilde{m}_i(\mathbf{q})\tilde{m}_j(-\mathbf{q})\left[\delta_{ij}\ \tilde{f}(q^2) + \frac{q_i q_j}{\varepsilon_0 q^2}\right]. \tag{S52}$$

To reduce the length of the following equations, we set $m_y^q(z) = 0$, i.e., we assume that the polarization modulation has vectorial components in the x and z directions only. Inserting Eq. (S51) into Eq. (S52) and performing the integrals over q_x and q_y, we obtain

$$\begin{aligned}\mathcal{F}_{\mathrm{pol}} = \frac{A}{2}\int \frac{\mathrm{d}q_z}{2\pi}&\left[\tilde{\overline{m}}(q_z)\tilde{\overline{m}}(-q_z)\left(\tilde{f}(q_z^2) + \varepsilon_0^{-1}\right)\right. \\ & + \tilde{m}_z^q(q_z)\tilde{m}_z^q(-q_z)\left(\tilde{f}(q_z^2 + \bar{q}_x^2) + \frac{q_z^2}{\varepsilon_0(q_z^2 + \bar{q}_x^2)}\right) \\ & \left. + \tilde{m}_x^q(q_z)\tilde{m}_x^q(-q_z)\left(\tilde{f}(q_z^2 + \bar{q}_x^2) + \frac{\bar{q}_x^2}{\varepsilon_0(q_z^2 + \bar{q}_x^2)}\right)\right].\end{aligned} \tag{S53}$$

After expansion of the non-electrostatic interaction kernel $\tilde{f}(q_z^2)$ to first order according to $\tilde{f}(q_z^2) \simeq \tilde{f}_0 + q_z^2\tilde{f}_1$, as done in Section IV, and back Fourier transformation, we obtain the Landau–Ginzburg model for a modulated polarization

profile

$$\frac{\beta \mathcal{F}_{\mathrm{LG}}}{A} = \int_{-d/2}^{d/2} \mathrm{d}z \left[a\overline{m}_z^2(z) + b\left(\nabla \overline{m}_z(z)\right)^2 \right.$$
$$+ a_z \left(m_z^q(z)\right)^2 + b_z\left(\nabla m_z^q(z)\right)^2$$
$$\left. + a_x \left(m_x^q(z)\right)^2 + b_x\left(\nabla m_x^q(z)\right)^2 \right], \qquad \text{(S54)}$$

which is a generalization of Eq. (1) in the main text. By comparison of Eq. (S53) and Eq. (S54) we obtain for the Landau–Ginzburg parameters $a_z = \beta(\tilde{f}_0 + \bar{q}_x^2 \tilde{f}_1)/2$, $b_z = \beta(\tilde{f}_1 + \varepsilon_0^{-1}\bar{q}_x^{-2})/2$, $a_x = \beta(\varepsilon_0^{-1} + \tilde{f}_0 + \bar{q}_x^2 \tilde{f}_1)/2$, $b_x = \beta(\tilde{f}_1 - \varepsilon_0^{-1}\bar{q}_x^{-2})/2$, and where a and b are given in Eq. (S25). We conclude from this short calculation that there is an entire spectrum of modulated order parameters which characterize the water polarization profile between two surfaces, each characterized by a different modulation wave vector $\bar{q}_x$ and described by a Landau–Ginzburg model with parameters a_x, a_z, b_x, b_z. Since these parameters depend on $\bar{q}_x$, also the decay length of the resulting hydration force will depend on $\bar{q}_x$, which might explain the simulation finding in the main text that different surfaces produce different decay lengths of the indirect hydration force. Importantly, each of these order parameters will make a contribution to the hydration pressure, whose magnitude depends on the coupling strength to the surface, to be discussed in future work.

XII. LATERAL DIPOLAR CORRELATIONS BETWEEN WATER MOLECULES

Here, we show that correlations of the lateral dipolar component between water molecules close to the two surfaces are significant and might be of importance for the resulting interaction between polar surfaces. To this end we study the decanol surface and assign water molecules to the decanols on the two opposing surfaces based on the shortest distance to the first carbon of decanol counting from the OH-group. Then, we compute the correlation for the dipolar Cartesian components $\alpha = x, y, z$,

$$\phi_\alpha = \left\langle P_{\mathrm{low},\alpha} P_{\mathrm{up},\alpha} \right\rangle / P_0^2, \qquad \text{(S55)}$$

where $\mathbf{P}_{\mathrm{low}}$ and $\mathbf{P}_{\mathrm{up}}$ are the dipole moments of the water molecules at opposing decanols at the lower and upper surface, respectively, and $P_0 = 0.49\,\mathrm{e\AA}$ is the dipole moment of a SPC/E water molecule.

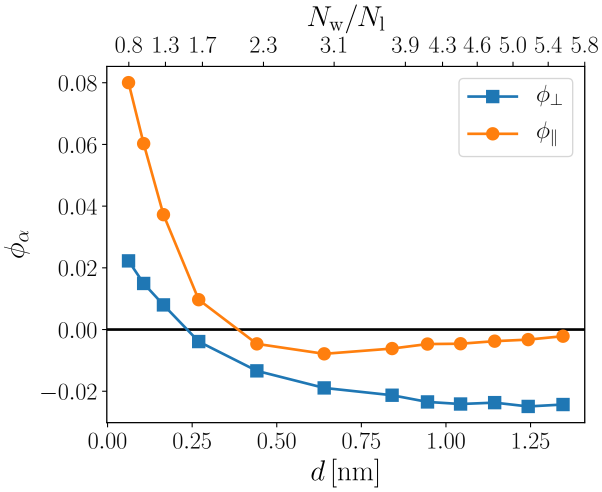

Figure S15. Correlation of the parallel and perpendicular dipolar components between water molecules located on the lower and upper decanol surfaces as defined by Eq. (S55).

Figure S15 shows the correlation of the dipole component perpendicular to the surfaces, $\phi_\perp = \phi_z$ (blue squares), and the correlation of dipole components parallel to the surfaces, $\phi_\parallel = \frac{1}{2}(\phi_x + \phi_y)$ (orange circles). As expected, the perpendicular dipole components are anticorrelated at large separations, reflecting the fact that the dipole orientation profile is antisymmetric. The correlation in fact turns positive in strong confinement at approximately $N_w/N_l < 1.5$

waters per lipid (upper x-axis in Fig. S15). The parallel component $\phi_{\parallel}$, shown as orange circles in Fig. S15, is also anticorrelated at large separations, which presumably is due to the preferred antiparallel orientation of the head groups, which minimizes their electrostatic energy [8]. We observe that the lateral correlations, $\phi_{\parallel}$, are of similar amplitude as the perpendicular correlations, $\phi_{\perp}$. As discussed in the main text and Section XI of the Supporting Information, lateral orientational correlations of water molecules are also expected to contribute to the hydration pressure and are missed when laterally averaging the water polarization profile.

---

[1] Mark James Abraham, Teemu Murtola, Roland Schulz, Szilárd Páll, Jeremy C. Smith, Berk Hess, and Erik Lindahl. GROMACS: High performance molecular simulations through multi-level parallelism from laptops to supercomputers. *SoftwareX*, 1–2:19–25, September 2015.

[2] Szilárd Páll, Mark James Abraham, Carsten Kutzner, Berk Hess, and Erik Lindahl. Tackling Exascale Software Challenges in Molecular Dynamics Simulations with GROMACS. In *Solving Software Challenges for Exascale*, pages 3–27. Springer, Cham, April 2014.

[3] Naveen Michaud-Agrawal, Elizabeth J. Denning, Thomas B. Woolf, and Oliver Beckstein. MDAnalysis: A toolkit for the analysis of molecular dynamics simulations. *J. Comput. Chem.*, 32(10):2319–2327, July 2011.

[4] Ulrich Essmann, Lalith Perera, Max L. Berkowitz, Tom Darden, Hsing Lee, and Lee G. Pedersen. A smooth particle mesh Ewald method. *The Journal of Chemical Physics*, 103(19):8577–8593, November 1995.

[5] H. J. C. Berendsen, J. R. Grigera, and T. P. Straatsma. The missing term in effective pair potentials. *J. Phys. Chem.*, 91(24):6269–6271, November 1987.

[6] Chris Oostenbrink, Alessandra Villa, Alan E. Mark, and Wilfred F. Van Gunsteren. A biomolecular force field based on the free enthalpy of hydration and solvation: The GROMOS force-field parameter sets 53a5 and 53a6. *Journal of Computational Chemistry*, 25(13):1656–1676, 2004.

[7] Matej Kanduč, Emanuel Schneck, and Roland R. Netz. Attraction between hydrated hydrophilic surfaces. *Chemical Physics Letters*, 610–611:375–380, August 2014.

[8] Emanuel Schneck, Felix Sedlmeier, and Roland R. Netz. Hydration repulsion between biomembranes results from an interplay of dehydration and depolarization. *PNAS*, 109(36):14405–14409, September 2012.

[9] Michael R. Shirts and John D. Chodera. Statistically optimal analysis of samples from multiple equilibrium states. *The Journal of Chemical Physics*, 129(12):124105, September 2008.

[10] D. P. Tieleman and H. J. C. Berendsen. Molecular dynamics simulations of a fully hydrated dipalmitoylphosphatidylcholine bilayer with different macroscopic boundary conditions and parameters. *The Journal of Chemical Physics*, 105(11):4871–4880, September 1996.

[11] O. Berger, O. Edholm, and F. Jähnig. Molecular dynamics simulations of a fluid bilayer of dipalmitoylphosphatidylcholine at full hydration, constant pressure, and constant temperature. *Biophysical Journal*, 72(5):2002–2013, May 1997.

[12] Erik Lindahl and Olle Edholm. Mesoscopic Undulations and Thickness Fluctuations in Lipid Bilayers from Molecular Dynamics Simulations. *Biophysical Journal*, 79(1):426–433, July 2000.

[13] Thomas Schubert, Emanuel Schneck, and Motomu Tanaka. First order melting transitions of highly ordered dipalmitoyl phosphatidylcholine gel phase membranes in molecular dynamics simulations with atomistic detail. *The Journal of Chemical Physics*, 135(5):055105, August 2011.

[14] Giovanni Bussi, Davide Donadio, and Michele Parrinello. Canonical sampling through velocity rescaling. *The Journal of Chemical Physics*, 126(1):014101, January 2007.

[15] Bartosz Kowalik, Thomas Schubert, Hirofumi Wada, Motomu Tanaka, Roland R. Netz, and Emanuel Schneck. Combination of MD Simulations with Two-State Kinetic Rate Modeling Elucidates the Chain Melting Transition of Phospholipid Bilayers for Different Hydration Levels. *The Journal of Physical Chemistry B*, 119(44):14157–14167, November 2015.

[16] Patrick S. Coppock and James T. Kindt. Determination of Phase Transition Temperatures for Atomistic Models of Lipids from Temperature-Dependent Stripe Domain Growth Kinetics. *J. Phys. Chem. B*, 114(35):11468–11473, September 2010.

[17] Jan Ulminus, Håkan Wennerström, Goran Lindblom, and Gosta Arvidson. Deuteron nuclear magnetic resonance studies of phase equilibriums in a lecithin-water system. *Biochemistry*, 16(26):5742–5745, 1977.

[18] C Grabielle-Madelmont and R Perron. Calorimetric studies on phospholipid—water systems. *Journal of Colloid and Interface Science*, 95(2):471–482, 1983.

[19] Matej Kanduč, Emanuel Schneck, and Roland R. Netz. Hydration Interaction between Phospholipid Membranes: Insight into Different Measurement Ensembles from Atomistic Molecular Dynamics Simulations. *Langmuir*, 29(29):9126–9137, July 2013.

[20] Alexandru Botan, Fernando Favela-Rosales, Patrick F. J. Fuchs, Matti Javanainen, Matej Kanduč, Waldemar Kulig, Antti Lamberg, Claire Loison, Alexander Lyubartsev, Markus S. Miettinen, Luca Monticelli, Jukka Määttä, O. H. Samuli Ollila, Marius Retegan, Tomasz Róg, Hubert Santuz, and Joona Tynkkynen. Toward Atomistic Resolution Structure of Phosphatidylcholine Headgroup and Glycerol Backbone at Different Ambient Conditions. *J. Phys. Chem. B*, 119(49):15075–15088, 2015.

[21] Sarah Lee, Alan Tran, Matthew Allsopp, Joseph B. Lim, Jérôme Hénin, and Jeffery B. Klauda. CHARMM36 United Atom Chain Model for Lipids and Surfactants. *J. Phys. Chem. B*, 118(2):547–556, January 2014.

[22] A. D. MacKerell, D. Bashford, M. Bellott, R. L. Dunbrack, J. D. Evanseck, M. J. Field, S. Fischer, J. Gao, H. Guo, S. Ha,

D. Joseph-McCarthy, L. Kuchnir, K. Kuczera, F. T. K. Lau, C. Mattos, S. Michnick, T. Ngo, D. T. Nguyen, B. Prodhom, W. E. Reiher, B. Roux, M. Schlenkrich, J. C. Smith, R. Stote, J. Straub, M. Watanabe, J. Wiórkiewicz-Kuczera, D. Yin, and M. Karplus. All-Atom Empirical Potential for Molecular Modeling and Dynamics Studies of Proteins. *J. Phys. Chem. B*, 102(18):3586–3616, April 1998.

[23] James C. Phillips, Rosemary Braun, Wei Wang, James Gumbart, Emad Tajkhorshid, Elizabeth Villa, Christophe Chipot, Robert D. Skeel, Laxmikant Kalé, and Klaus Schulten. Scalable molecular dynamics with NAMD. *J. Comput. Chem.*, 26(16):1781–1802, 2005.

[24] LJ Lis, M. McAlister, N. Fuller, RP Rand, and VA Parsegian. Interactions between neutral phospholipid bilayer membranes. *Biophysical journal*, 37(3):657, 1982.

[25] J F Nagle. Area/lipid of bilayers from NMR. *Biophys J*, 64(5):1476–1481, May 1993.

[26] Horia I. Petrache, Steven W. Dodd, and Michael F. Brown. Area per Lipid and Acyl Length Distributions in Fluid Phosphatidylcholines Determined by 2h NMR Spectroscopy. *Biophysical Journal*, 79(6):3172–3192, 2000.

[27] T. J. McIntosh, A. D. Magid, and S. A. Simon. Steric repulsion between phosphatidylcholine bilayers. *Biochemistry*, 26(23):7325–7332, November 1987.

[28] K Gawrisch, D Ruston, J Zimmerberg, V A Parsegian, R P Rand, and N Fuller. Membrane dipole potentials, hydration forces, and the ordering of water at membrane surfaces. *Biophys J*, 61(5):1213–1223, May 1992.

[29] Bartosz Kowalik, Alexander Schlaich, Matej Kanduč, Emanuel Schneck, and Roland R. Netz. Hydration Repulsion Difference between Ordered and Disordered Membranes Due to Cancellation of Membrane–Membrane and Water-Mediated Interactions. *J. Phys. Chem. Lett.*, pages 2869–2874, June 2017.

[30] Alexander Schlaich, Ernst W. Knapp, and Roland R. Netz. Water Dielectric Effects in Planar Confinement. *Phys. Rev. Lett.*, 117(4):048001, July 2016.

[31] Herbert Fröhlich. *Theory of dielectrics: dielectric constant and dielectric loss.* Clarendon Press, 1958.

[32] Douwe Jan Bonthuis, Stephan Gekle, and Roland R. Netz. Dielectric Profile of Interfacial Water and its Effect on Double-Layer Capacitance. *Phys. Rev. Lett.*, 107(16):166102, October 2011.

[33] Philippe A. Bopp, Alexei A. Kornyshev, and Godehard Sutmann. Static Nonlocal Dielectric Function of Liquid Water. *Phys. Rev. Lett.*, 76(8):1280–1283, February 1996.

[34] M. Rami Reddy and M. Berkowitz. The dielectric constant of SPC/E water. *Chemical Physics Letters*, 155(2):173–176, February 1989.

[35] Philip Loche, Cihan Ayaz, Amanuel Wolde-Kidan, Alexander Schlaich, and Roland R. Netz. Universal and Nonuniversal Aspects of Electrostatics in Aqueous Nanoconfinement. *J. Phys. Chem. B*, 124(21):4365–4371, May 2020.

[36] Philip Loche, Laura Scalfi, Mustakim Ali Amu, Otto Schullian, Douwe J. Bonthuis, Benjamin Rotenberg, and Roland R. Netz. Effects of surface rigidity and metallicity on dielectric properties and ion interactions at aqueous hydrophobic interfaces. *J. Chem. Phys.*, 157(9):094707, September 2022.

[37] M. G. Cottam, D. R. Tilley, and B. Zeks. Theory of surface modes in ferroelectrics. *J. Phys. C: Solid State Phys.*, 17(10):1793, 1984.

[38] Rudolf Podgornik and Boštjan Žekš. Hydration force and hydration regulation. *Studia Biophysica*, 111(2-3):135–142, 1986.

[39] Gregor Cevc, Rudi Podgornik, and Boštjan Žekš. The free energy,enthalpy and entropy of hydration of phospholipid bilayer membranes and their difference on the interfacial separation. *Chemical Physics Letters*, 91(3):193–196, September 1982.

[40] Alexander Schlaich, Bartosz Kowalik, Matej Kanduč, Emanuel Schneck, and Roland R. Netz. Physical mechanisms of the interaction between lipid membranes in the aqueous environment. *Physica A: Statistical Mechanics and its Applications*, 418:105–125, January 2015.

[41] Matej Kanduč, Alexander Schlaich, Emanuel Schneck, and Roland R. Netz. Hydration repulsion between membranes and polar surfaces: Simulation approaches versus continuum theories. *Advances in Colloid and Interface Science*, 208:142–152, June 2014.

[42] Alexander Schlaich, Bartosz Kowalik, Matej Kanduč, Emanuel Schneck, and Roland R. Netz. Simulation Techniques for Solvation-Induced Surface-Interactions at Prescribed Water Chemical Potential. In Godehard Sutmann, Johannes Grotendorst, Gerhard Gompper, and Dominik Marx, editors, *Computational Trends in Solvation and Transport in Liquids*, volume 28 of *IAS Series*, pages 155–185. Forschungszentrum Jülich GmbH, Jülich, March 2015.

[43] B. Widom. Some Topics in the Theory of Fluids. *The Journal of Chemical Physics*, 39(11):2808–2812, December 1963.

[44] In-Chul Yeh and Max L. Berkowitz. Ewald summation for systems with slab geometry. *The Journal of Chemical Physics*, 111(7):3155–3162, August 1999.
